# Functional Characterization of Soybean *Glyma04g39610* as a Brassinosteroid Receptor Gene and Evolutionary Analysis of Soybean Brassinosteroid Receptors

**DOI:** 10.3390/ijms17060897

**Published:** 2016-06-07

**Authors:** Suna Peng, Ping Tao, Feng Xu, Aiping Wu, Weige Huo, Jinxiang Wang

**Affiliations:** 1 The State Key Laboratory for Conservation and Utilization of Subtropical Agro-Bioresources, South China Agriculture University, Guangzhou 510642, China; pengsuna2013@163.com (S.P.); hntaoping@163.com (P.T.); fengxu@hybribio.cn (F.X.); wuaiping1@163.com (A.W.); 18819266123@163.com (W.H.); 2 College of Agriculture & Root Biology Center, South China Agricultural University, Guangzhou 510642, China

**Keywords:** brassinosteroids, BR receptor, soybean, gene family, evolution

## Abstract

Brassinosteroids (BR) play important roles in plant growth and development. Although BR receptors have been intensively studied in *Arabidopsis*, the BR receptors in soybean remain largely unknown. Here, in addition to the known receptor gene *Glyma06g15270* (*GmBRI1a*), we identified five putative BR receptor genes in the soybean genome: *GmBRI1b*, *GmBRL1a*, *GmBRL1b*, *GmBRL2a*, and *GmBRL2b*. Analysis of their expression patterns by quantitative real-time PCR showed that they are ubiquitously expressed in primary roots, lateral roots, stems, leaves, and hypocotyls. We used rapid amplification of cDNA ends (RACE) to clone *GmBRI1b* (*Glyma04g39160*), and found that the predicted amino acid sequence of GmBRI1b showed high similarity to those of AtBRI1 and pea PsBRI1. Structural modeling of the ectodomain also demonstrated similarities between the BR receptors of soybean and *Arabidopsis*. GFP-fusion experiments verified that GmBRI1b localizes to the cell membrane. We also explored *GmBRI1b* function in *Arabidopsis* through complementation experiments. Ectopic over-expression of *GmBRI1b* in *Arabidopsis* BR receptor loss-of-function mutant (*bri1-5 bak1-1D*) restored hypocotyl growth in etiolated seedlings; increased the growth of stems, leaves, and siliques in light; and rescued the developmental defects in leaves of the *bri1-6* mutant, and complemented the responses of BR biosynthesis-related genes in the *bri1-5 bak1-D* mutant grown in light. Bioinformatics analysis demonstrated that the six BR receptor genes in soybean resulted from three gene duplication events during evolution. Phylogenetic analysis classified the BR receptors in dicots and monocots into three subclades. Estimation of the synonymous (*K*_s_) and the nonsynonymous substitution rate (*K*_a_) and selection pressure (*K*_a_/*K*_s_) revealed that the *K*_a_/*K*_s_ of BR receptor genes from dicots and monocots were less than 1.0, indicating that BR receptor genes in plants experienced purifying selection during evolution.

## 1. Introduction

The brassinosteroid (BR) phytohormones play important roles in many aspects of plant growth and development, including root growth and development [1,2], stomatal development [3], seed germination [4], skotomorphogenesis [5,6], phototropism [7], nodulation [8], immunity responses [9], and abiotic stress responses [10,11]. As early as 1990, scientists reported that BR promoted adventitious rooting in soybean hypocotyl cuttings [12]. Later work showed that BR can promote stem growth in soybean [13] and the application of BR increased soybean tolerance to drought by increasing the concentrations of soluble sugars and proline [14]. Additionally, BR increases the expression of soybean *SAUR 6B*, which promotes epicotyl elongation in a time-dependent manner [13]. These studies indicate that BRs regulate soybean growth, development, and stress responses at a physiological and molecular level.

Work in *Arabidopsis* and rice has revealed the BR signaling pathway. BR signals are detected by BR receptors such as BRASSINOSTEROID INSENSITIVE 1 (BRI1) in the cell membrane [15,16]. In the absence of BRs, BRI1 is bound by the membrane-localized BRI1 KINASE INHIBITOR 1 (BKI1) [17]. Upon perception of BR, BRI1 disassociates from BKI1 [17] and interacts with BRI1-ASSOCIATED RECEPTOR KINASE 1 (BAK1), a membrane kinase and co-receptor of BRI1 [18]. BRI1 and BAK1 *trans*-phosphorylate each other [17]. BRASSINOSTEROID INSENSITIVE 2 (BIN2), the downstream regulator of BRI1, is a highly conserved GSK kinase and a negative regulator of BR signaling; BIN2 phosphorylates the transcription factors BRASSINAZOLE-RESISTANT 1 (BZR1) and BZR2 thus inactivating them [19,20,21,22]. In contrast, PROTEIN PHOSPHATASE 2A (PP2A) mediates the dephosphorylation, and thus activation of BZR1 [23]. High levels of BR in plants leads to the inactivation of BIN2. The dephosphorylated BZR1 and BZR2 shuttle from the cytoplasm to the nucleus and bind to the promoters of numerous downstream genes, thus strengthening BR signaling [24]. BIN2 and BZR1 are regulated by proteasome-dependent pathways in *Arabidopsis* [21,25].

AtBRI1, the most important BR receptor in *Arabidopsis*, has a membrane-localization signal peptide in the N-terminus, 25 leucine-rich repeat (LRR) domains, and a 70-amino acid island between LRR XXI and LRR XXII [26], which is indispensable for the perception of BR [27,28]. Although BAK1 does not directly bind to BR and only has five LRR motifs, BAK1 promotes BR signaling by interacting with and phosphorylating BRI1. Two recent structural biology studies have shown that AtBRI1 is a BR receptor [27,28]. These studies revealed that brassinolide (BL) binds to a highly hydrophobic surface groove on BRI1 (LRR) and the ectodomain is crucial for the binding of BR [27,28]. This insight could be extrapolated to investigate BR receptors in agricultural plants.

Identifying BR receptors in other plants and deciphering their functions provides an important initial step toward deciphering BR signaling networks and understanding their evolution. *Arabidopsis* has three functional BR receptors, BRI1, BRL1, and BRL3. AtBRL2 appears to be non-functional in BR signaling [29,30]. The rice genome contains four BR receptor genes, *OsBRI1*, *OsBRL1*, *OsBRL2*, and *OsBRL3* [31,32]. BR receptors have also been identified in tomato [33], pea [34], barley [35], cotton [36], maize [37], and wheat [38]. Although the BR signaling pathway has been well studied in *Arabidopsis* and rice, it is not well understood in soybean. Recent work reported that soybean *Glyma06g12570* encodes a functional BR receptor [39]. Considering the high levels of duplication in the soybean genome [40], we postulated that soybean may have other functional BR receptors.

In this study, we conducted an evolutionary and functional examination of soybean BR receptors. Including the known gene *Glyma06g15270* [39], we identified six BR receptor genes in the soybean genome and analyzed their expression patterns. We also further examined one gene, *Glycine max Glyma04g39610* (*GmBRI1b*), which encodes a homolog of AtBRI1. GmBRI1b localizes to the membrane and can function as a BR receptor in *Arabidopsis*. Analysis of the evolution of BR receptors in plants showed that BR receptors were subjected to purifying or negative selection.

## 2. Results

### 2.1. Isolation of Glyma04g39610 (GmBRI1b)

To clone soybean brassinosteroid receptors, we used the AtBRI1 protein sequence to search the soybean EST database [41], using the BLASTP algorithm. We found that the amino acid sequence encoded by a tentative contig (TA51665) showed a high similarity to a region of AtBRI1. Therefore, we used the contig to design 5′-RACE and 3′-RACE primers to amplify the flanking regions of TA51665. After cloning and sequencing the flanking region, a cDNA fragment approximately 4 kb long containing a poly (A) tail was obtained (data not shown). Using ORF finder [42], the full cDNA was predicted to contain a long open reading frame (Genbank Accession No. KU360113) that encodes a protein of 1187 amino acids. Alignment with the soybean genome sequence indicated that this protein is encoded by *Glyma04g39610* [43]. As *Glyma06g15270* (*GmBRI1*) was reported to encode a BR receptor [39], we named *Glyma04g39610* as *GmBRI1b* and renamed *Glyma06g15270* as *GmBRI1a*. Further bioinformatics analysis showed that GmBRI1b contains a membrane-localized signal peptide in the N-terminus followed by 25 LRRs, a transmembrane domain, and a Ser/Thr kinase domain in the C-terminus (Table 1 and Table S1). Alignment analysis indicated that GmBRI1b has 69% and 81% identity to AtBRI1 and pea BRI1 (PsBRI1), respectively (Figure S1).

As reported, *AtBRI1* and rice *OsBRI1* lack introns [15,31]. To determine whether *GmBRI1b* contains introns, we designed primers covering the initiation codon and stop codon and amplified the genomic DNA. After sequencing, we found that *GmBRI1b* also lacks introns. This indicated that the structure of the BR receptor genes has been highly conserved between these two species.

### 2.2. Identification of Other BR Receptor Genes in Soybean

Given that a whole-genome duplication occurred during soybean evolution [40], we proposed that *Glycine max* has additional BR receptor genes. Thus, using the released soybean genome from 2010 [40], we performed a BLAST search against the soybean genome [43] using the BLASTP algorithm using the sequences of the four Arabidopsis BR receptors as queries. Apart from *GmBRI1a* [39] and *GmBRI1b*, four additional putative BR receptor genes were found, *Glyma04g12860* (*GmBRL1a*), *Glyma06g47870* (*GmBRL1b*), *Glyma05g26771* (*GmBRL2a*), and *Glyma0809750* (*GmBRL2b*) on chromosomes 4, 5, 6, and 8, respectively (Table 1). All six soybean BR receptors contain a kinase domain (KD) and five out of the six have a signal peptide (SP) and a transmembrane domain (TM) as predicted by the SMART program [44] (Table 1).

GmBRI1a, GmBRI1b, GmBRL1b, and GmBRL2b were predicted to be membrane proteins via the PSORT program [45], and GmBRL1a and GmBRL2a appeared to be localized in the cytoplasm and nucleus, respectively. In addition, five of the BR genes had no introns, but *GmBRL2a* had one intron (Table 1).

Next, we aligned the full amino acid sequences of the BR receptor proteins from *Arabidopsis*, rice, soybean, tobacco, potato, *Medicago*, and barley. As shown in Figure S1, the similarities between the BR receptors were as high as 80%, indicating that the BR receptors evolved slowly in higher plants. Recent structural studies indicated that the ectodomain in BR receptors is the BR-binding domain [27,28]. Thus, we compared the amino acid sequences of the ectodomains of the BR receptors from soybean, *Arabidopsis*, rice, barley, pea, and tomato. As expected, the similarities were high over 77% (Figure S2). Additionally, the KDs among the BR receptors from the different species were also highly conserved (data not shown), indicating the importance of KDs for BR function.

In *Arabidopsis*, the island domain (ID) between LLRS XXI and XXII has been reported to be involved in BR binding [27,28]. To determine whether the soybean BR receptors have the ID, we aligned the sequences with their counterparts in *Arabidopsis* and other species. As shown in Figure S3, the sequences of the IDs are highly conserved in BR receptors among the different species, though we did observe that the ID sequences of GmBRI2a and GmBRI2b showed more variation than those of GmBRI1a, GmBRI1b, GmBRL1a, and GmBRL1b.

To evaluate the duplication of the BR receptor genes in soybean, we used the PGDD software [46]. Three BR receptor gene duplication events were detected in soybean, *GmBRI1a* VS *GmBRI1b*, *GmBRL1a* VS *GmBRL1b*, and *GmBRL2a* VS *GmBRL2b* (Figure S4).

### 2.3. Transcript Levels of GmBRI1b and Other BR Receptor Genes in Soybean

**Figure 1:**
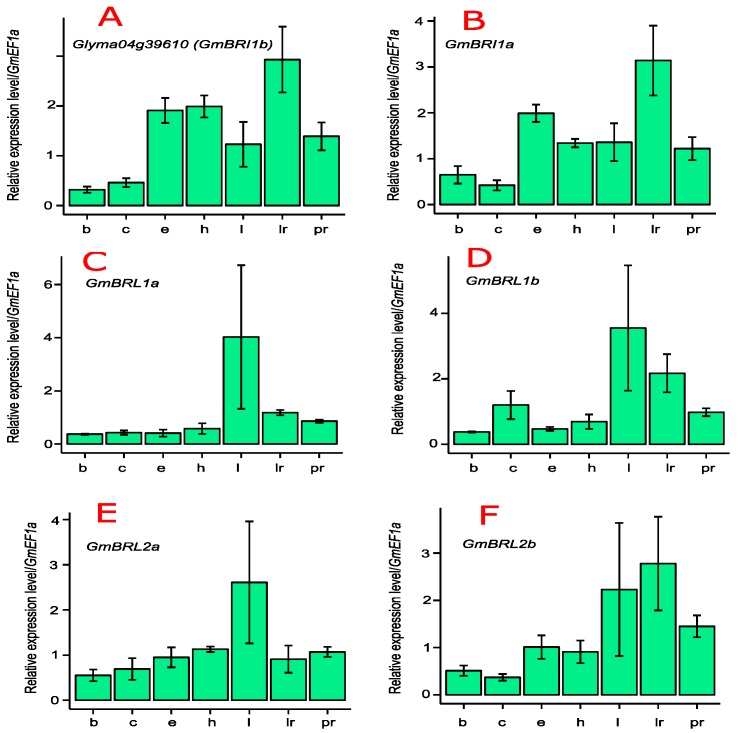

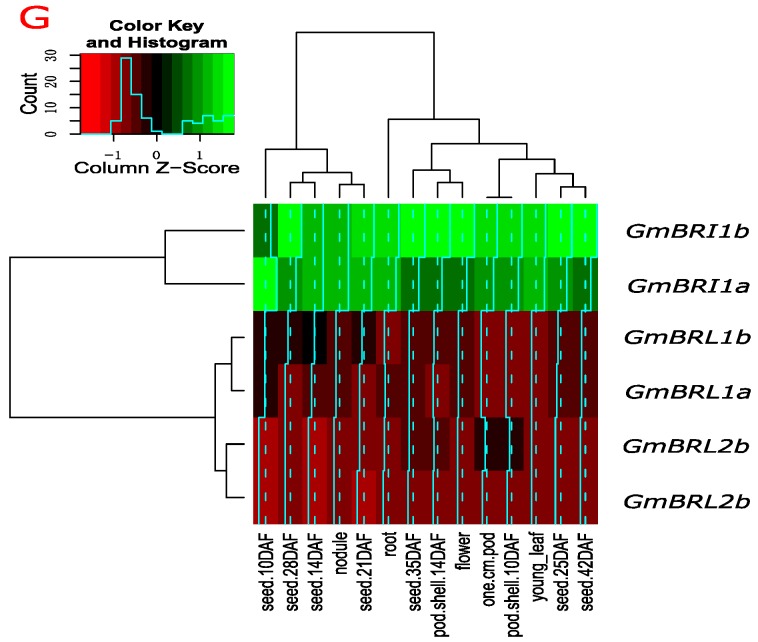
Expression of soybean BR receptors:*GmBRI1b* (**A**); *GmBRI1a* (**B**); *GmBRL1a* (**C**); *GmBRIL1b* (**D**); *GmBRL2a* (**E**); and *GmBRL2b* (**F**) in apical buds (b), cotyledons (c), epicotyls (e), hypocotyls (h), leaves (l), lateral roots (lr), and primary roots (pr). *GmEF1a* was used to normalize the qRT-PCR data. Results in (**A**–**F**) were means ± SD from three independent experiments, each of which were technically repeated three times. The normalized RNA-Seq expression data of soybean BR receptor genes were downloaded from SoyBase [47] (**G**).

We used quantitative real-time PCR (qRT-PCR) to determine the expression patterns and transcript abundance of *GmBRI1b* in soybean. *GmBRI1b* was universally expressed in the primary roots, lateral roots, hypocotyls, epicotyls, cotyledons, apical buds, and leaves of soybean (Figure 1A).

The transcript levels of other *Glycine max* BR receptors from different organs were also determined through qRT-PCR. As shown in Figure 1B, the expression pattern of *GmBRI1a* was similar to that of *GmBRI1b* (Figure 1A) and the abundances of both were higher in lateral roots, as was *GmBRL2b* (Figure 1F). This indicates their important roles in lateral root development. Similarly, the transcript levels of *GmBRL1a* (Figure 1C), *GmBRL1b* (Figure 1D), and *GmBRL2a* (Figure 1E) were relatively higher in leaves.

Additionally, we investigated the expression levels of the BR receptors in soybean based on previous RNA-Seq studies [47]. As shown in Figure 1G, the transcript levels of *GmBRI1a* and *GmBRI1b* were relatively high in young leaf, flower, pod, seed, root, and nodule tissues, indicating their important roles in soybean growth and development. *GmBRL1a* and *GmBRL1b* were also expressed in all tested organs although their transcript levels were lower than those of *GmBRI1a* and *GmBRI1b*. By contrast, *GmBRI2a* and *GmBRI2b* had relatively low transcript levels in seeds and nodules (Figure 1G). The relatively high expression levels of *GmBRI1a* and *GmBRI1b* in nodules suggest their important roles in nodulation. Based on similarities of their expression patterns, the six soybean BR receptor genes can be classified into three groups, *GmBRI1a* and *GmBRI1b*; *GmBRL1a* and *GmBRL1b*; and *GmBRI2a* and *GmBRI2b*.

### 2.4. Subcellular Localization of GmBRI1b

**Figure 2:**
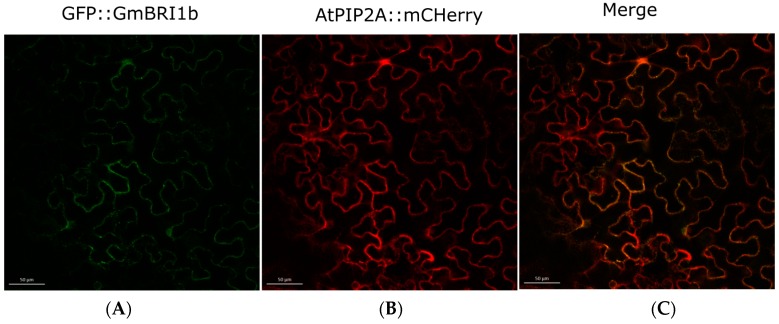
Subcellular localization of GmBRI1b. The subcellular localization was determined with the constructs GFP::GmBRI1b: (**A**) GFP::BRI1b; (**B**) AtPIP1A::mCherry; and (**C**) merged image. The GFP and mCherry signals were detected at 484 and 544 nm, respectively. Scale bar = 50 µm.

As mentioned above, a signal peptide in the N-terminus and a transmembrane (TM) domain were predicted in GmBRI1b [44] (Table 1). This indicated that GmBRI1b might be a cell membrane protein. To determine the subcellular localization of GmBRI1b, we constructed a fusion protein of GmBRI1b::GFP using the gateway vector pMDC43. We then co-transformed tobacco leaf epidermal cells with constructs encoding GmBRI1b::GFP and the plasma membrane marker protein AtPIP2A::mCherry [48]. Using laser confocal microscopy, we detected fluorescence signals only in the plasma membrane and the GFP fluorescence co-localized with mCherry fluorescence (Figure 2). This suggested that GmBRI1b is a cell membrane protein.

### 2.5. Functional Analysis of GmBRI1b in Arabidopsis

BRs increase cell elongation in higher plants and a deficiency of BR results in smaller, curled leaves and shorter petioles [15]. The BR receptor AtBRI1 plays crucial roles in *Arabidopsis* growth and development, especially in stem and leaf growth. We hypothesized that GmBRI1b can function as a BR receptor to promote stem and leaf growth. To test this hypothesis and investigate the function of *GmBRI1b*, we tested whether *GmBRI1b* could complement the *Arabidopsis BRI1* loss-of-function mutant *bri1-5 bak1-1D* [49]. The *bri1-5* allele contains a Tyr-69 substitution at the first cysteine pair of AtBRI1 that appears to be important for its dimerization [49]. The *bak1-1D* line, in which expression of *BAK1* is activated by an insertion of four tandem copies of the cauliflower mosaic virus (CaMV) 35S promoter, has stronger expression of *BAK1*, compared with wild type [18]. In contrast to the *bri1-5* mutant, the *bri1-5 bak1-1D* mutant has a relatively higher stature and longer petioles, but still exhibits a deficiency in BR signaling, represented by relatively shorter stems and petioles relative to the Ws-2 wild type [18].

**Figure 3:**
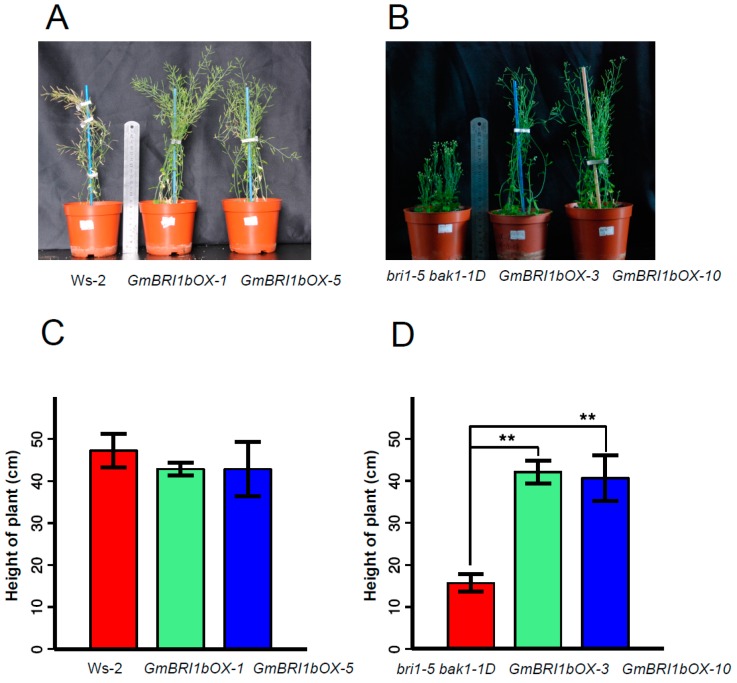
Over-expression of *GmBRI1b* increased plant height in the *bri1-5 bak1-1D* mutant. The height of 50-day-old Ws-2 wild type and two corresponding *GmBRI1b* over-expression lines (*GmBRI1b-OX*) (**A**,**C**); and the *bri1-5 bak1-1D* mutant and corresponding *GmBRI1b-OX* lines (**B**,**D**). Results are means ± SD from five plants. Experiments were repeated two times with similar trend (Student’s *t*-test, ** *p* < 0.01).

To test whether *GmBRI1b* can complement the *Arabidopsis* mutants, we created transgenic *GmBRI1b* over-expression lines (*GmBRI1b-OX*) driven by CaMV 35S promoter in the Ws-2 wild-type plants and in the *bri1-5 bak1-1D* mutant (Figure S5A,B). After 50 days, we measured the height of the wild-type plants, *bri1-5 bak1-1D* mutant, and the over-expression lines grown under the same lighting and temperature conditions. Over-expression of *GmBRI1b* had little effect on the height of the transgenic Ws-2 wild-type plants (Figure 3A,C). As reported, the *bri1-5 bak1-1D* mutant was shorter than the wild-type Ws-2 plants [18] (Figure 3B,D), but over-expression of *GmBRI1b* restored the normal plant height in the transgenic *bri1-5 bak1-1D* mutant. For example, *GmBRI1b* over-expression lines of the *bri1-5 bak1-1D* mutant were 2.6× and 2× taller compared with the non-transformed mutant (Figure 3B,D), further supporting the hypothesis that GmBRI1b functions as a BR receptor.

**Figure 4:**
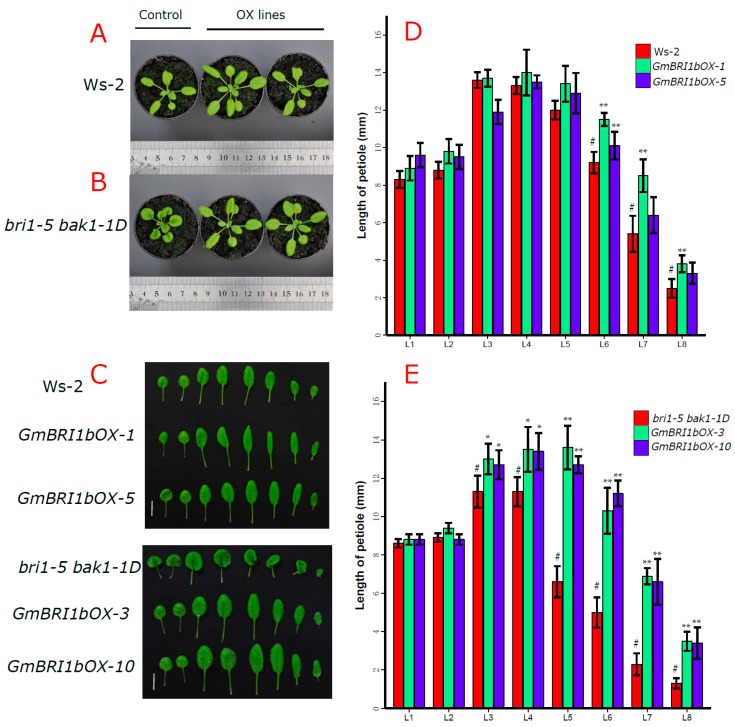
Ectopic over-expression of *GmBRI1b* increased the length of the petioles in the transgenic *bri1-5* *bak1-1D* mutant and wild type Ws-2. The 25-day-old plants (**A**). The 1st to the 8th leaves of the Ws-2 wild type and two corresponding *GmBRI1b* over-expression lines (*GmBRI1b-OX*) (**B**); and the *bri1-5 bak1-1D* mutant and corresponding *GmBRI1b-OX* lines (**C**). The length of the petioles from the 1st to the 8th leaves (L1-8) was measured in 25-day-old seedlings of the Ws-2 wild-type lines (**D**); and the *bri1-5 bak1-1D* mutant lines (**E**). Results are means ± SD from five independent experiments (in total, 25 seedlings were measured) (#, control; Student’s *t*-test, * *p* < 0.05; ** *p* < 0.01). Scale bar = 1 cm.

We also observed leaf and petiole growth and development in the Ws-2 wild type, and the *bri1-5 bak1-1D* mutant, and their corresponding *GmBRI1b* over-expression lines. The *bri1-5 bak1-1D* mutant had smaller leaves and shorter petioles compared to the Ws-2 wild type at 25 days after germination (Figure 4A), but over-expression of *GmBRI1b* in the transgenic *bri1-5 bak1-1D* mutant resulted in narrower leaves and longer petioles than in the non-transformed mutant (Figure 4A–C). Over-expression of *GmBRI1b* significantly increased the length of the 6th, 7th, and 8th leaf petiole in the Ws-2 wild type plants (*p* < 0.01), but no differences were found in the 1st to 5th leaves (Figure 4D). Over-expression of *GmBRI1b* in the transgenic *bri1-5* *bak1-1D* mutant significantly increased elongation of the 3rd to the 8th leaves (*p* < 0.05 or *p* < 0.01), but not the 1st and the 2nd leaves (Figure 4E).

**Figure 5:**
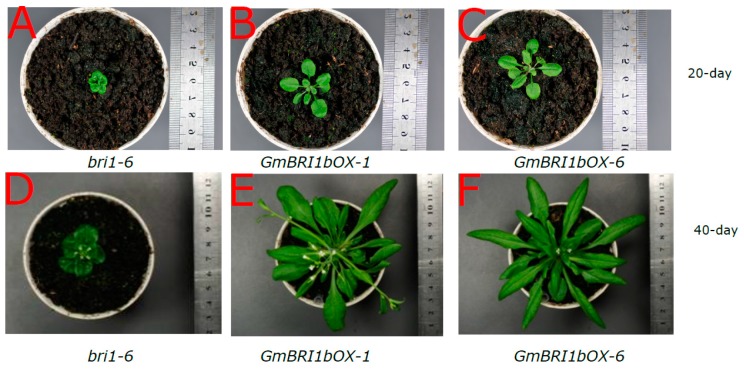
Ectopic over-expression of *GmBRI1b* restored the wild-type leaf phenotype in the transgenic *bri1-6* mutant. Leaf phenotypes of the 20-day-old *bri1-6* mutant (**A**); and the two corresponding *GmBRI1b* over-expression lines *GmBRI1bOX-1* (**B**) and *GmBRI1bOX-6* (**C**); Leaf phenotypes of the 40-day-old *bri1-6* mutant (**D**); and the two corresponding *GmBRI1b* over-expression lines *GmBRI1bOX-1* (**E**) and *GmBRI1bOX-6* (**F**).

The *bri1-6* mutant has smaller, curled leaves with very short petioles. Ectopic over-expression of *GmBRI1b* in the transgenic *bri1-6* mutant (Figure S5C) significantly increased petiole length and restored the normal wild-type leaf phenotypes in 20-day-old (Figure 5A–C) and 40-day-old (Figure 5D–F) plants.

**Figure 6:**
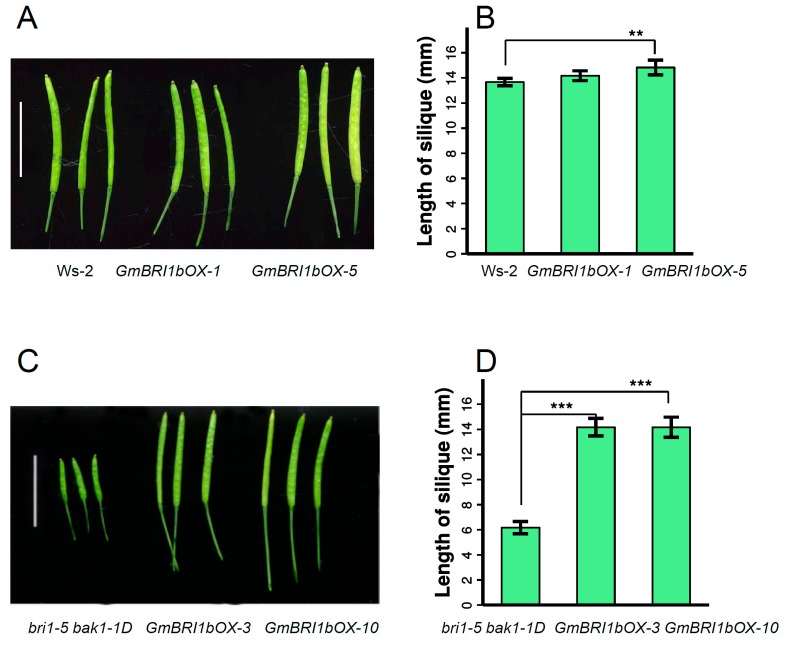
Over-expression of *GmBRI1b* increased the length of the siliques in the *bri1-5bak1-1D* mutant. The length of siliques in the Ws-2 wild type and the two corresponding *GmBRI1b* over-expression lines (*GmBRI1b-OX*) (**A**,**B**); and the *bri1-5 bak1-1D* mutant and corresponding *GmBRI1b-OX* lines (**C**,**D**). Results are means ± SD from three independent experiments (a total of 15 seedlings were measured) (Student’s *t*-test, ** *p* < 0.01, *** *p* < 0.001). Scale bar = 1 cm.

In *Arabidopsis,* limitations in BR levels or defects in BR signaling lead to shorter siliques [50]. The length of siliques in the same position and the same developmental stage were measured in the shoots of the Ws-2 wild type, and *bri1-5 bak1-1D* mutant, and their corresponding *GmBRI1b* over-expression lines. For the Ws-2 wild-type lines, the siliques of *GmBRI1bOX-5* were significantly longer than those of the non-transformed Ws-2 wild type, but this was not true for *GmBRI1bOX-1* (Figure 6A,B). For the *bri1-5 bak1-1D* lines, over-expression of *GmBRI1b* significantly increased the length of the siliques (Figure 6C,D).

Collectively, these results demonstrate that *GmBRI1b* functions as a BR receptor at the physiological and genetic level.

### 2.6. Ectopic Over-Expression of GmBRI1b Increased the Hypocotyl Length of the bri1-5 bak1-1D Mutant and Changed the Responses of the Wild Type and bri1-5 bak1-1D Mutant to Brassinazole

Brassinazole (Brz) effectively inhibits BR biosynthesis [51] and Brz treatment decreases the growth of etiolated *Arabidopsis* hypocotyls [51,52]. To evaluate the effects of ectopic over-expression of *GmBRI1b* on the response to Brz, *Arabidopsis* seeds were germinated in half-strength MS media and then transferred to different concentrations of Brz-containing MS media after three days. After six days, the lengths of the hypocotyls were measured.

**Figure 7:**
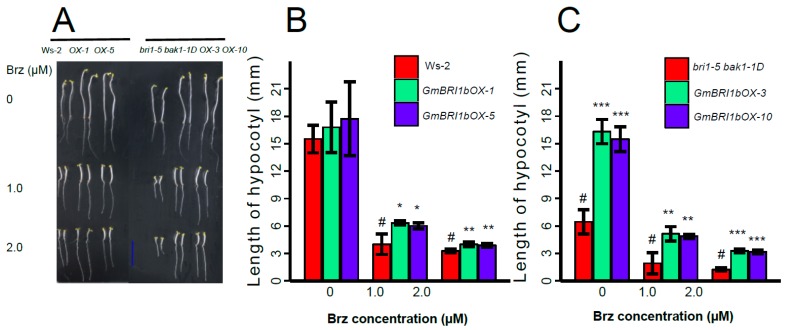
Over-expression of *GmBRI1b* increased the tolerance to Brz in the *Arabidopsis* plants. Hypocotyl measurements were taken after exposure to different concentrations of Brz in seedlings of the Ws-2 wild type and the two corresponding *GmBRI1b* over-expression lines (*GmBRI1b-OX*) (**A**,**B**); and the *bri1-5 bak1-1D* mutant and corresponding *GmBRI1b-OX* lines (**A**,**C**). All seedlings were grown under full darkness for seven days. Results are means ± SD from three independent experiments (a total of 30 seedlings were measured) (#, control; Student’s *t*-test, * *p* < 0.05; ** *p* < 0.01; *** *p* < 0.001). Scale bar = 1 cm.

The differences in hypocotyl lengths between the dark-grown Ws-2 wild-type plants and the two corresponding over-expression lines were not significant under the control conditions (no Brz treatment). In plants treated with 1 µM Brz, the hypocotyl lengths of the two over-expression lines were 1.58× and 1.50× longer than those of the non-transgenic Ws-2 wild-type seedlings (*p* < 0.05). In plants treated with 2 µM Brz, the lengths of the hypocotyls in the two over-expression lines were 1.23× and 1.19× longer than those of the non-transgenic Ws-2 wild-type seedlings (*p* < 0.01) (Figure 7A,B).

Ectopic over-expression of *GmBRI1b* in the transgenic *bri1-5 bak1-1D* mutant promoted hypocotyl growth in dark-grown seedlings in the untreated and Brz-treated conditions (Figure 7A,C). In the untreated plants, the hypocotyl lengths of the two over-expression lines were increased by 1.53× and 1.40× over those of the non-transformed *bri1-5 bak1-1D* mutant. In the plants treated with 1 µM Brz, over-expression of *GmBRI1b* increased the length of the hypocotyls by 1.68× and 1.54× (*p* < 0.01). In the plants treated with 2 µM Brz, the length of the hypocotyls in the over-expression lines increased by 1.61× and 1.53× (*p* < 0.01) compared with those of the *bri1-5 bak1-1D* mutant lacking the transgene (Figure 7C).

Taken together, these data demonstrated that ectopic over-expression of *GmBRI1b* decreased the sensitivity of the Ws-2 wild type and the *bri1-5 bak1-1D* mutant to exogenous Brz by restoring hypocotyl growth. These results further suggest that GmBRI1b functions as a BR receptor in *Arabidopsis*.

### 2.7. Over-Expression of GmBRI1b Altered the Expression Level of BR Biosynthesis-Related Genes in the bri1-5 bak1-1D Mutant

**Figure 8:**
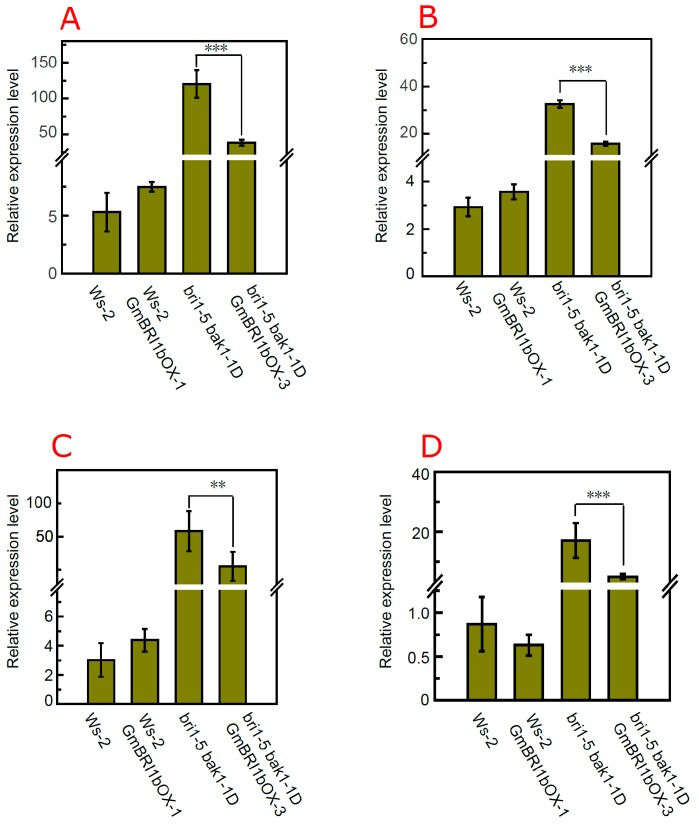
Effect of ectopic over-expression of *GmBRI1b* on the expression of BR biosynthesis-related genes in the transgenic *bri1-5 bak1-1D* mutant. Seedlings were grown as described in Materials and Methods. qRT-PCR was used to detect the relative expression levels of: *DWF4* (**A**); *CPD* (**B**); *BR6ox-1* (**C**); and *BR6ox-2* (**D**) in Ws-2 and *bri1-5 bak1-1D* mutant and their corresponding over-expression lines. Results are means ± SD from three independent experiments with three technical replicates (Student’s *t*-test, ** *p*< 0.01, *** *p*< 0.001).

Previous studies have revealed that the expression levels of BR biosynthesis-related genes, such as *DWF4*, *CPD*, *BR6ox-1*, and *BR6ox-2*, are regulated by negative feedback by BR itself and by BR signaling [53,54,55]. Therefore, we selected these four marker genes to explore the effects of ectopic over-expression of *GmBRI1b* on the crosstalk between BR signaling and BR biosynthesis at the molecular level. Over-expression of *GmBRI1b* had little effect on *DWF4* transcription in wild type Ws-2, but significantly down-regulated transcription of *DWF4* in the transgenic *bri1-5 bak1-1D* mutant (Figure 8A). Dislike Ws-2, ectopic over-expression of *GmBRI1b* significantly repressed the expression of *CPD* in the *bri1-5 bak1-1D* over-expression line (*p* < 0.001, Figure 8B). Over-expression of *GmBRI1b* repressed the expression of *BR6ox-1* in the *bri1-5 bak1-1D* over-expression line by 0.51× compared with the non-transformed *bri1-5 bak1-1D* mutant (Figure 8C). In addition, over-expression of *GmBBRI1b* repressed the expression of *BR6ox-2* by 0.29× in the *bri1-5 bak1-1D* over-expression line compared with their corresponding non-transformed mutant, but this is not true in wild type (Figure 8D). Thus, these results indicate that ectopic over-expression of *GmBRI1b* enhanced BR signaling in the *bri1-5 bak1-1D* mutant, and that GmBRI1b is functional in *Arabidopsis*.

### 2.8. Structural Modeling of Soybean BR Receptors

Two studies reported the X-ray diffraction structure of the AtBRI1 ligand-binding domain (ectodomain) in 2011 [27,28]. In AtBRI1, a 70-amino-acid ID between LRR XXI and XXII, which folds back into the interior of the super helix, generates a pocket for binding brassinolide [27,28]. A 69 amino acid long ID was found between the LRR XXI and XXII in GmBRI1b (Table S1 and Figure S3). Computational homology modeling is a powerful tool to investigate conservation between homologous proteins across plant species [56]. After searching the PDB database [57], 3RGZ and 3RGX were selected as the best templates with which to rebuild the structure of soybean BR receptors. In addition, we also reconstructed AtBRL1, AtBRL2, and AtBRL3. As shown in Table S2, 3RGZ and 3RGX were chosen as the best templates for GmBRL1a, GmBRL1b, GmBRL2a, GmBRL2b, AtBRL1, and AtBRL3, or GmBRI1a and GmBRI1b, respectively. The related parameters in Table S2 indicated the reliability of the homology modeling.

**Figure 9:**
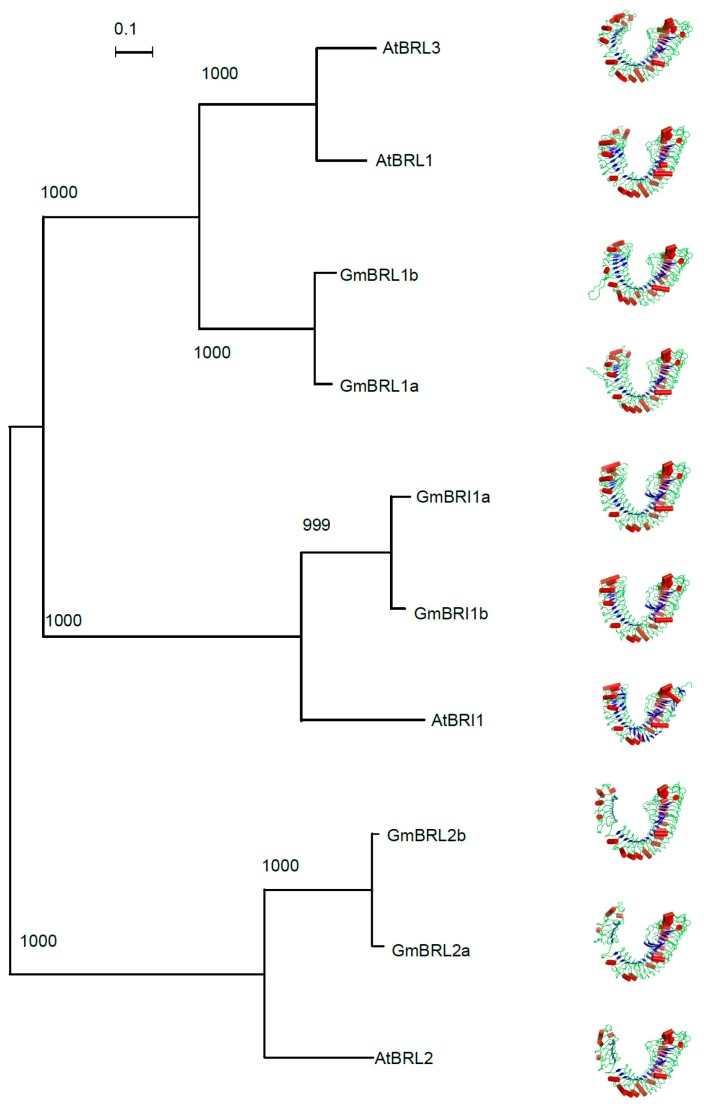
Structural modeling of six soybean BR receptors. The program PhyML3.0 [58] was used to reconstruct the phylogenetic tree of BR receptors from *Arabidopsis* and soybean. The evolutionary lineages of four BR receptors in *Arabidopsis* and six BR receptors in soybean were compared. The LG model for amino acid substitutions with estimated Gamma distribution was used to reconstruct the tree and the bootstrap value was set as 1000. A total of ten BR receptors were classified into Clades I, II, and III. The numbers above each branch of the tree are the bootstrap values. Scale bar indicates 0.1 amino acid substitution over evolution. In addition, the ectodomains of ten BR receptors were modeled with MODELLER9.11 software [59] based on the template of AtBRI1, which was determined with X-ray crystallization on a 3-D level in 2011 [28]. The PDB files were processed with PyMOL v1.5 software [60].

The α-helix and β-sheet were found in the ectodomain of soybean BR receptors (Figure 9). The structural models of the *Glycine max* BR receptors and *Arabidopsis* BRL1 and BRL3 show high similarities in the 3-D structures of the ectodomains of the BR receptors. The tertiary structures of the BR receptors in each class also show high similarities (Figure 9). This suggested that the protein structures of BR receptors are conserved across plants.

### 2.9. Evolutionary Analysis of BR Receptors in Plants

**Figure 10:**
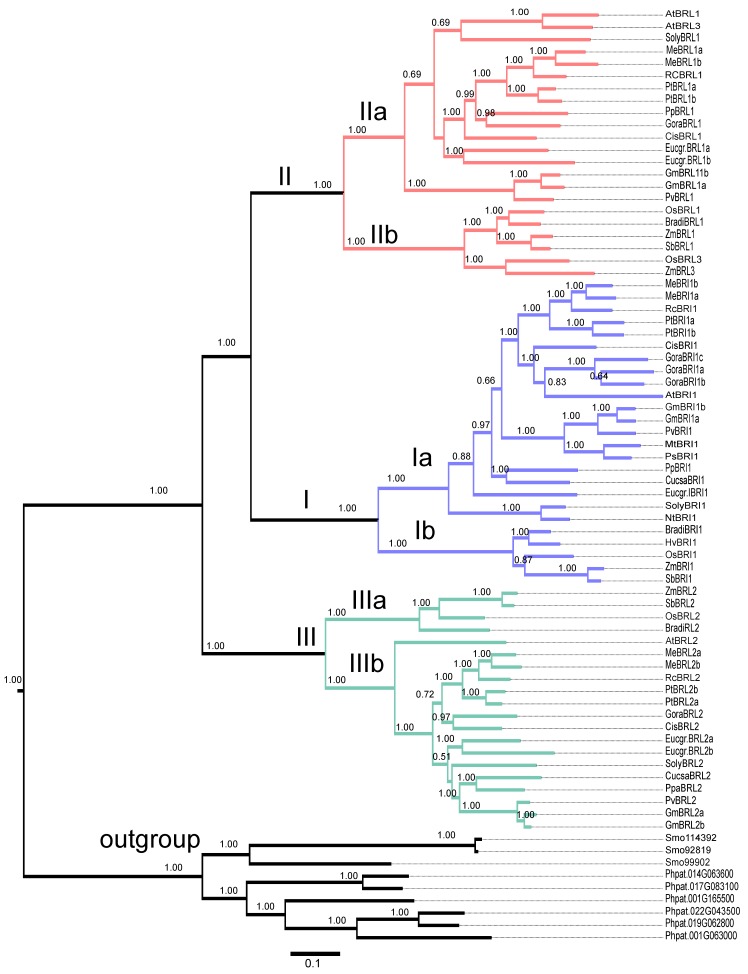
Evolutionary analysis of BR receptors in plants. BLASTP was used to search for BR receptor homologs in different plant species. A total of 76 BR receptors as shown in Spreadsheet S1 were analyzed from monocots (*Oryza sativa* (Os), *Zea mays* (Zm), *Sorghum bicolor* (Sb), *Brachypodium* *distachyon* (Bradi)), dicots (*Arabidopsis thaliana* (At), *Glycine max* (Gm), *Solanum* *lycopersicum* (Soly), *Medicago* *truncatula* (Mt), *Phaseolus vulgaris* (Pv), *Populus* *trichocarpa* (Pt), *Eucalyptus grandis* (Eucgr), *Citrus* *sinensis* (Cis), *Gossypium* *raimondii* (Gora), *Cucumis sativa* (Cusa), *Prunus* *persica*(Pp), *Manihot* *esculenta* (Me), *Ricinus* *communis* (Rc), and *Nicotiana* *tabacum* (Nt)), moss (*Physcomitrella patens* (Phpat)), and fern (*Selaginella* *moelledorffii* (Smo)) and grouped into three clades, CladesI, II, and III. Additionally, nine BR receptor homologs from moss and fern were determined to be members of an outgroup. The values above the branches are the probability of the bootstrap value with 1000 repeats. MrBayes 3.2 software was used to reconstruct the phylogenetic tree as described in the Methods Section. Scale bar indicates 0.1 amino acid substitution over evolution.

To analyze the evolutionary relationship among BR receptors across plant species, including receptors from moss, ferns, gymnosperms, and angiosperms, we collected BR receptor sequences using BLASTP searches. First, we performed BLAST searches against different plant genomes with the amino acid sequences of AtBRI1, AtBRL1, AtBRL2, and AtBRL3. We then selected the proteins with high scores as candidate BR receptors in the different plant species. Last, we performed domain analysis with the SMART program and predicted the kinase domain and LRR domains. Based on these criteria, the sequences of 76 putative BR proteins from *Physcomitrella patens*, *Selaginella moelledorffii*, four monocots, three legumes (*Glycine max*, *Medicago truncatula*, and *Phaseolus vulgaris*), and 11 dicots were aligned with ClustalW 2.1. Next, we reconstructed the phylogenetic tree of the BR receptors with MrBayes 3.2 software [61]. Three proteins from *Physcomitrella patens* and six from *Selaginella moelledorffii* were classified into the same subgroup with 100% bootstrap support (Figure 10) and were considered to be an outgroup in the reconstructed phylogenetic tree. The remaining 67 BR receptor proteins from monocots and dicots were grouped into Clades I, II, and III (100% bootstrap support). In each clade, the proteins from the monocots (rice, maize, sorghum, and *Brachypodium distachyon*) or from the dicots formed well-separated subclades with 100% bootstrap support (Figure 10). The branch length indicates the history of evolution.

The three clades were represented by BRI1, BRL1/BRL3, and BRL2. As mentioned above, BRL2 in rice and *Arabidopsis* appeared to be non-functional. Two *Glycine max* BR receptors (GmBRL2a and GmBRL2b) also seemed to have no function in BR signaling. When compared with the evolutionary distance of the BR receptors from legumes and other dicots, the BR receptors from legumes showed more conservation than other BR receptors.

In addition, we also reconstructed the phylogenetic tree with the Maximum Likelihood (ML) method to reconstruct the evolutionary relationship among BR receptors with PhyML [58]. The phylogenetic tree generated with this method was the same as that generated from the Bayesian method (data not shown).

**Figure 11:**
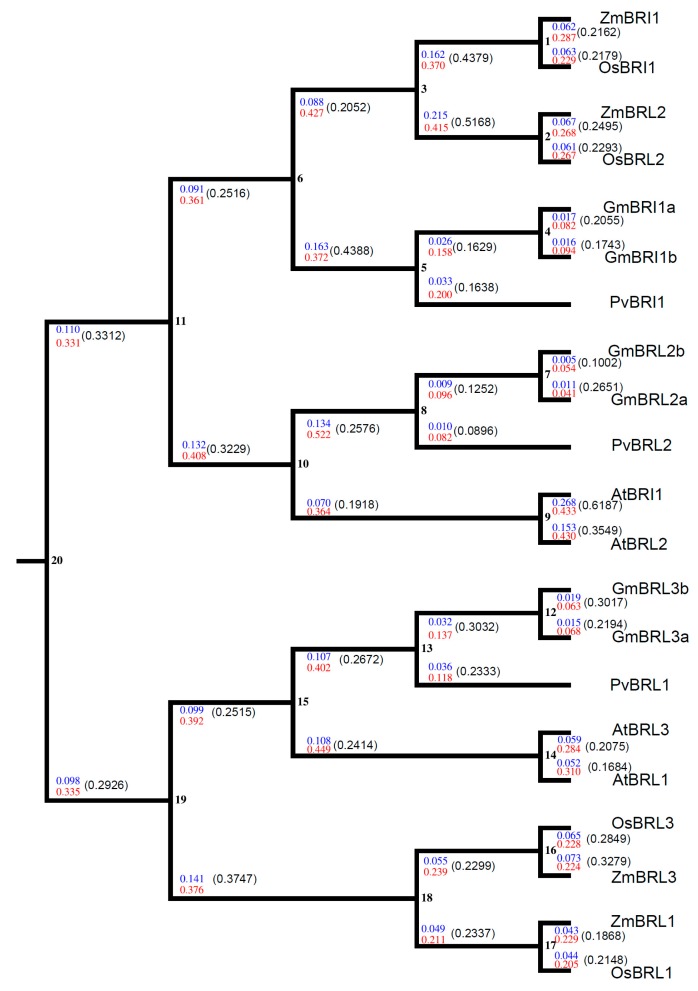
Estimation of *K*_a_/*K*_s_ in the BR receptor genes from soybean, common bean, *Arabidopsis,* rice, and maize. The cDNA sequences and amino acid sequences of the BR receptors from dicots (soybean, common bean, and *Arabidopsis*) and monocots (rice and maize) were used to estimate the *K*_a_/*K*_s_. Twenty nodes are shown. The *K*_a_ and *K*_s_ values in each node and branch are marked in blue and red, respectively. The *K*_a_/*K*_s_ in each branch is indicated in parentheses and all *K*_a_/*K*_s_ values were less than 1.0.

We investigated the synonymous (*K*_s_) and nonsynonymous substitution rate (*K*_a_) and selection pressure (*K*_a_/*K*_s_) of the BR receptor genes in higher plants during evolution. The aligned BR receptor amino acid sequences and their corresponding cDNA sequences that were conserved across soybean, rice, maize, *Arabidopsis*, and common bean were analyzed using the *K*_a_/*K*_s_ calculator [62]. As shown in Figure 11, the *K*_a_/*K*_s_ values in all nodes and branches were less than 1.0, indicating that BR receptors were subjected to strong selection pressure.

## 3. Discussion

Although BR signaling has been extensively studied in rice and *Arabidopsis*, BR signaling in soybean is still largely unknown. In this study, we cloned the soybean BR receptor gene *Glyma04g39610* (*GmBRI1b*) and demonstrated that it functions as a BR receptor in *Arabidopsis* at the physiological, genetic, and molecular levels. In addition to this BR receptor gene and another known soybean BR receptor gene *Glymg06g15270* [39], we identified four other soybean BR receptor genes.

BRs play important roles in plant growth, development, and stress adaptations such as cell elongation and division, seed germination, photomorphogenesis and skotomorphorgenesis, responses to salts and heavy metals, and pathogen resistance. BRs have been found in 61 species of embryophytes, including 53 angiosperms, six gymnosperms, one pteridophyte (*Equisetum arvense*), and one bryophyte (*Marchantia polymorpha*) [63]. Additionally, two single-celled green freshwater algae (*Chlorophyta*; *Chlorella vulgaris* and *Hydrodictyon reticulatum*) and the marine brown alga *Cystoseira myrica* biosynthesize BRs [64]. This indicates that BRs appear to be conserved phytohormones in plants. Considering that BRs even exist in single-celled plants, the identification of BR receptor-like proteins in moss and fern may deepen our understanding of the evolution of BR signaling in plants. Our phylogenetic analysis implies that BR receptors exist universally throughout the plant kingdom (Figure 10).

Similar to previously reported BR receptor genes in Arabidopsis and rice [15,31], *GmBRI1b* does not contain introns. We determined that at least six BR receptor genes exist in the soybean genome, in contrast to only four BR receptor genes in both *Arabidopsis* and rice, indicating that BR signaling may be more complex in soybean. In addition, two soybean BR receptor genes, *GmBRL2a* and *GmBRL2b*, showed a close evolutionary relationship with *AtBRL2* and *OsBRL2*, which have been reported to have no function in BR signaling [29,32]. The role of soybean *GmBRI2a* and *GmBRI2b* in BR signaling needs further study.

As previously reported, functional BR receptors in *Arabidopsis* and rice are localized in cell membranes [31,65]. A signal peptide was found in the N-terminus of GmBRI1b (Table 1). GFP fusion experiments showed that GmBRI1b localizes in the plasma membrane (Figure 2). This supports the idea that GmBRI1b perceives BR at the cell membrane.

*AtBRI1* and *OsBRI1* are ubiquitously expressed in all tissues [31,65]. We found that *GmBRI1b* is expressed in apical buds, cotyledons, epicotyls, hypocotyls, leaves, lateral roots, and primary roots (Figure 1A), implying a crucial and universal role in soybean growth and development. The soybean RNA-Seq data [47] supported our results (Figure 1G). Moreover, the transcript abundance of two soybean BR receptor genes, *GmBRI1a* and *GmBRI1b*, in nodules was relatively high, suggesting that these two genes play important roles in nodulation (Figure 1G). Application of Brz on mature leaves or into the culture media increased the nodule number and inhibited internode growth in the soybean cultivar Enrei and foliar applications of BR inhibited nodulation and root growth in the super-nodulating mutant *En6500*, indicating the existence of BR signaling modules in soybean [66]. Moreover, these results indicate that endogenous BR homeostasis or BR signaling might control nodulation. Recent studies demonstrated that pea BR biosynthesis mutant (*lk*), and BR receptor mutant (*lkb*) had fewer lateral roots and nodules and the decreased nodule number did not seem to be attributed to changes in endogenous GA or auxin levels [8]. Therefore, the roles of BR receptors in legume nodulation need further study.

In our study, we noticed differences in expression among the soybean BR receptors. For instance, the expression levels of *GmBRL2a* and *GmBRL2b* were very low in seeds, but the levels of *GmBRI1a* and *GmBRI1b* were higher in seeds (Figure 1G). We also found higher expression of *GmBRI1a* and *GmBRI1b* in flowers (Figure 1G), indicating that these genes might regulate flower and seed development. The highest expression of *GhBRI1* was found in hypocotyls, while its transcripts were much lower in mature roots [36]. *GmBRI1a* was found to be highly expressed in soybean hypocotyls and is up-regulated by exogenous BR [39]. We detected higher expression of *GmBRI1b* in hypocotyls and lateral roots in fourteen-day-old soybean seedlings, in which active cell proliferation and elongation are occurring. A previous study showed that some gene pairs resulting from gene duplication in soybean showed similar expression patterns during nodulation, while others showed different expression patterns [67]. Interestingly, we found that three BR receptor gene pairs generally showed similar transcription patterns (Figure 1A–F). As three gene duplication events occurred during soybean evolution (Figure S4), it is possible that the promoter sequences in the three gene pairs are similar and some common *cis*-elements might be found among them.

The loss of function mutation in BR signaling or biosynthesis genes in Arabidopsis results in a dwarf phenotype and late flowering [6,68,69,70]. We found that ectopic over-expression of *GmBRI1b* in transgenic *bri1-6*, and *bri1-5 bak1-1D* mutant restored the normal wild-type phenotype, including the height of the seedlings and the length of petioles and siliques, leaf growth. Thus, we concluded that *GmBRI1b* functions as a BR receptor in *Arabidopsis* at the physiological and genetic level. We also demonstrated that GmBRI1b acts as a functional BR receptor in *Arabidopsis* at the molecular level. Ectopic over-expression of *GmBRI1b* repressed the relatively high expression of *DWF4*, *CPD*, *BR6OX-1*, and *BR6OX-2* in the transgenic *bri1-5 bak1-1D* mutant. GmBRI1a was classified into the same subclade with GmBRI1b in this study. In a previous study, when *GmBRI1a* was expressed in the *Arabidopsis bri1-5* mutant, reversed the developmental defects of the *bri1-5* mutant [39], although the subcellular localization of GmBRI1a was not determined [39]. The BR-binding activity of GmBRI1b remains unknown. Results from this study combined with previous studies suggest that GmBRIa and GmBRI1b are BR receptors.

Domain analysis showed that GmBRI1b contains an N-terminal signal peptide, a transmembrane domain, a kinase domain, and 25 LRR motifs (Table 1 and Table S1). This is in accordance with the structures of AtBRI1 and GmBRI1a [39]. We noticed that in higher plants, the complete BR receptor domains evolved through two domain-gain events in the ancestral receptor-like kinase, the juxtamembrane domain (JM) and the island domain (ID) [71]; the JM domain was acquired during the early diversification of plants and the ID domain formed in the ancestors of angiosperms and gymnosperms after their divergence from moss [71].

Based on structural biology studies conducted in 2012 [27,28], the ectodomain is responsible for the binding of the BR receptor to brassinolide. As expected, the highly conserved ectodomain sequences of the soybean BR receptors with BR receptors of other plant species were observed (Figure S2). Moreover, our structural modeling indicates that the ectodomain of all six soybean BR receptors form similar tertiary structures to that of AtBRI1 (Figure 9). This indicates that the ectodomain of BR receptors in *Glycine max* might have an identical function to that of AtBRI1 *in vivo*.

The amino acid sequences in the IDs between different plant species are highly conserved and IDs participate directly in BR binding [27,38]. The results in Figure S3 show that the ID in soybean BR receptors is highly conserved, but the ID in GmBRL2a andGmBRL2b show more variation than the other four soybean BR receptors. GmBRL2a and GmBRL2b were classified into the same subclade with AtBRL2, which have been documented to have no function in BR signaling [29]. This raises the question of whetherGmBRL2a and GmBRL2b play a role in BR signaling, and if no, can this be ascribed to the differences in the IDs? Of note, IDs only exist in gymnosperms (conifers and gnetophytes) and angiosperms. In contrast, the more ancient plant LRR–KD domain configuration generated from green algae after the split between the green algae and red algae [71].

We investigated the evolution of BR receptors in plants. BLAST searches indicated that a similar kinase existed in lower plants such as *Physcomitrella patens* and *Selaginella moelledorffii*. This is consistent with a previous study that suggested that the kinase domain (KD) is more ancient [71]. When we reconstructed the phylogenic tree, we found that the proteins from *Physcomitrella patens* and *Selaginella moelledorffii* had a close relationship, although we did not identify conserved BR receptors in *Chlamydomonas reinhardtii*. Considering the existence of BRs in single-celled plants [64], we can not rule out the existence of BR receptor-like proteins in single-celled plants as some receptor kinase proteins might act as BR receptors. Interestingly, Cheon *et al.* [72] reported that *Selaginella* lacks a homolog of AtBRI1, but does have downstream proteins such as BIN2, BSU1, and BZR1. This implies that the BR receptor complex evolved in a common ancestor of lycophytes, gymnosperms, and angiosperms. We found that a total of 67 BR receptors from dicots and monocots can be evolutionarily grouped into three clades represented by AtBRI1/OsBRI1, AtBRL1/OsBRL1, and AtBRL2/OsBRL2 with significant bootstrap support, which is in accordance with a previous study [72]. Each clade can be divided into two subclades, Ia, Ib, IIa, IIb, IIIa, and IIIb (100% bootstrap support), one subclade from monocots, and the other from dicots (Figure 10). These results indicate that three ancestral BR receptor genes might generate a plethora of BR genes in plants. One subgroup, which was represented by AtRBL2, might have lost its BR receptor function during evolution [29], but the reason for this is currently unknown.

We analyzed the selection pressure of BR receptors in plants during evolution. Notably, in each clade, the *K*_a_/*K*_s_ was less than 1.0 (Figure 11). This suggests that the BR receptor genes in higher plants were subjected to negative or purifying selection during evolution and indicates that the amino acid residues in BR receptor proteins are important and that a nonsynonymous mutation would be lethal or harmful for species survival. A previous study showed that the *D*_n_/*D*_s_ values were less than 1.0 in all gene pairs and detected no positive selection during BR receptors evolution [71]. Similarly, the substitution ratio of non-synonymous to synonymous SNPs (*K*_a_/*K*_s_) of as high as 76% analyzed *JAZ* genes across 13 monocot and dicot species was less than 1.0 [73]. Additionally, the *K*_a_/*K*_s_ of the squamosa promoter binding protein (SBP)-box genes that encodes crucial transcription factors in plants were less than 0.5 [74]. Our results are in accordance with previous reports that the *K*_a_/*K*_s_ of some important genes in multiple plant species are less than 1.0 [75].

In the case of soybean, BR receptors have undergone duplication due to whole-genome duplication events [40]. The soybean genome duplication also makes it much more difficult to identify BR-insensitive mutants. The CRISPR-Cas9 tool, together with artificial microRNA to knock out or knock down genes, would be valuable to further study other soybean BR receptor genes and for investigations of BR receptor functions in soybean.

## 4. Materials and Methods

### 4.1. Plant Materials and Growth

*Glycine max* cultivar BD2 was used as the soybean material. One week after germination, soybean plants were cultured with half-strength Hoagland’s solution in the greenhouse. The wild type *Arabidopsis* Ws-2, and the *bri1-5* *bak1-D* and *bri1-6* mutants were used. Seeds were stratified in the dark at 4 °C for 2 days, then surface-sterilized for 30 s in 75% ethanol followed by 8–10 min in 10% NaClO solution and washed five times with sterilized distilled water, then plated on half-strength MS media containing 1% sucrose and 0.8% agar with a pH of 5.8. Plates were kept in a growth chamber with a 16-h light/8-h dark cycle and a temperature cycle of 23 °C light/21 °C dark. After one week, seedlings were transplanted into soil and cultured as indicated above.

### 4.2. Extraction of Genomic DNA and RNA and Reverse Transcription of mRNA

Soybean and *Arabidopsis* genomic DNA and RNA were extracted with the CTAB and TRIzol methods, respectively. The cDNAs were reverse transcribed through MLV-transcriptase according to the vendor’s instructions. Other molecular experimental procedures were based on standard methods.

### 4.3. RACE Cloning of GmBRI1b

Two-week-old soybean BD2 seedlings were used to extract total RNA with the TRIzol method. Reverse transcription was carried out following standard methods to obtain cDNA. We used the SMART RACE kit (Takara Biomedical Technology, Beijing, China) to clone the *GmBRI1b* cDNA 5′ fragments and 3′fragments. Specific primer pairs were designed with PerlPrimer [76] and are listed in Table S3.

### 4.4. Analysis of Expression Patterns of Soybean BR Receptor Genes

We designed specific primer pairs with PerlPrimer [76] based on the cDNA sequences and genomic sequences [43] of the six soybean BR receptors to detect the expression levels of the six genes in different organs. Seven-day-old seedlings of the soybean cultivar BD2 germinated on sands were transferred to half-strength Hoagland’s nutrient solution. Seven days later, the roots, stems, and leaves were sampled for extraction of total RNA. Quantitative real-time PCR (qRT-PCR) was used to test the expression levels of the six BR receptor genes. The PCR thermal cycler parameters used were 40 cycles of 95 °C for 15 s, 60 °C for15 s, and 72 °C for 30 s. *GmEF1a* (*Glyma19g07240*) was used to normalize the expression levels. We used Rotorgen software and absolute quantification method [77] to calculate the PCR results with the amplicon of *GmEF1a* as standard. The data presented were from three independent biological experiments.

### 4.5. Over-Expression of GmBRI1b in Arabidopsis

The plasmid pCHF3 (a gift from Christian Fankhauser) was digested with *EcoRI* and *SalI* to release the CaMV 35S constitutive promoter and then the 35S promoter was ligated with the *EcoRI* and *SalI*-digested plasmid pPZP221 (a gift from Jianming Li). We named this plasmid p35SPZP221. We amplified *GmBRI1b*cDNA, which contains *SalI* and *SmaI* restriction sites, with primer pairs. Next, we ligated *GmBRI1b* with plasmid p35SPZP221 digested with *SalI* and *SmaI*. After sequencing, *Arabidopsis* wild-type Ws-2, the *bri1-5 bak1-1D* and *bri1-6* mutants were transformed with *Agrobacterium* GV3101 via the floral dipping method [78]. Then, the transgenic seedlings were screened in half-strength MS media that was solidified with 0.8% agar and contained 100 mg/L gentamycin. The homozygous, one-copy insertion transgenic lines were confirmed with PCR and the χ^2^-test and the qualifying over-expression lines were used for further experiments.

### 4.6. Subcellular Localization of GmBRI1b

To determine the subcellular localization of GmBRI1b, we constructed the fusion protein of GmBRI1b with GFP. The ORF of *GmBRI1b*, in which no stop codon exists, was amplified by PCR using the primers 5′-GGGGACAAGTTTGTACAAAAAAGCAGGCTTCATGAAAGCTCTGTACAGAAGCT-3′ and 5′-GGGGACCACTTTGTACAAGAAAGCTGGGTCCTAATGCTTGCTCAATTCAGGG-3′. The amplified cDNA fragment was then recombined into the pMDC43 vector, thus producing the GFP::BRI1b construct under the control of the 35S promoter. AtPIP2A, which was shown to be a plasma membrane aquaporin [48], fused with mCherry was used as the plasma membrane marker protein. *Agrobacterium tumefaciens* mediated transient expression in *Nicotiana* *benthamiana* tobacco leaves was conducted as described [79] with minor modifications. The *Agrobacterium* GV3101 strain harboring the constructs of GFP::GmBRI1b and AtPIP2A-mCherry [56] was inoculated in YEP medium with the appropriate antibiotics and incubated for 16 h with shaking at 28 °C. After centrifugation at 5000 rpm for 10 min, the cell pellet was re-suspended to OD_600_ = 1.0 in the infiltration medium (10 mM MgCl_2_, 10 mM MES, and 150 µM acetosyringone). The cell suspension was then allowed to standing at 22 to −24 °C for 2 to 3 h before infection into the tobacco leaves. A mix of cells containing the same quantities of *GFP::GmBRI1b* and *AtPIP2A-mCherry*, were then infiltrated into the leaves of three- to four-week-old tobacco plants. After three days, we observed the fluorescence distribution in the tobacco epidermal cells at 488 nm (GFP) and 587 nm (mCherry) wave lengths by confocal laser scanning microscopy (LSM780, Zeiss, Jena, Germany).

### 4.7. Phenotypic Analysis of Transgenic Arabidopsis

For sterilized solid media culture, seeds were sterilized as described above and sown in solid half-strength MS medium containing 0, 1, or 2 µM Brz. Then, the square petri dishes were wrapped with double layers of foil and were placed vertically in full darkness. After 7 days, the seedlings were scanned and analyzed with ImageJ software to quantify the length of the hypocotyls.

For determination of height, silique length, and petiole length, the seeds of the wild-type Ws-2, the *bri1-6* and *bri1-5 bak1-1D* mutants, and their corresponding over-expression lines were stratified at 4 °C for 2 days to break dormancy and then sown in soil. After the culture period, the height of the plants and the length of the petioles and siliques were measured.

### 4.8. Determination of the Expression Levels of BR Biosynthesis-Related Genes in the Wild Type, the Mutant, and Their Corresponding over-Expression Lines

To determine the expression levels of the BR biosynthesis-related genes *CPD*, *DWF4*, *BR6ox-1*, and *BR6ox-2*, seedlings of one-week-old Ws-2 wild type, and *bri1-5 bak1-1D* mutant, and the over-expression lines were transplanted into soil for one month under standard growth conditions. Then, whole plants were used to extract total RNA with the TRIzol method. cDNAs were obtained by reverse transcriptase reactions. qRT-PCR was used to determine the transcript abundances of *CPD*, *DWF4*, *BR6ox-1*, and *BR6ox-2*. *AtEF-1a* was used as a reference gene to normalize the qRT-PCR results. The qRT-PCR data were determined by absolute quantification method [77] with the amplicon of AtEF-1a as standard. Specific primer pairs are listed in Table S3.

### 4.9. Determination of the GmBRI1b Structure

Based on the full sequence of the *GmBRI1b* cDNA, we designed primers at the regions of the initiation codon and the stop codon to amply the genomic DNA fragment. After sequencing, we compared the genomic and cDNA sequences of *GmBRI1b* using SIM4 software [80] to determine the protein structure of GmBRI1b.

### 4.10. Alignment of BR Receptors

T-coffee software [81] was used to align the sequences of the full BR receptor, ectodomain, and island domain of the BRs from the different plant species *Glycine max*, *Arabidopsis thaliana*, *Solanum* *lycopersicum*, *Oryza* *sativa*, *Pisum* *sativum*, *Hordeum* *vulgare*, and *Medicago* *truncatula*.

### 4.11. Structural Modeling of Soybean BR Receptors

The sequences of the ectodomain of AtBRI1, AtBRL1, AtBRL3, and the six soybean BR receptors were aligned with T-coffee v11.0 [81]. We then searched for the best-scoring templates in the Protein Data Bank [57] and selected 3RGXZ and 3RGXA. Next, we rebuilt nine structural models (six soybean BR receptors, AtBRL1, AtBRL3, and AtBRL2) of each ectodomain using Modeller v9.10 [56,82] and reported the results using the best model based on the DOPE score [83]. The PDB files and images were processed with PyMOL v1.5 [60].

### 4.12. Estimation of Selection and Substitution Rates

The cDNA and amino acid sequences of the BR receptors from the monocots rice and maize and the dicots soybean, common bean, and *Arabidopsis* were used to calculate nonsynonymous (*K*_a_) and synonymous (*K*_s_) substitution rates and their ratio (*K*_a_/*K*_s_) for each node/branch via a *K*_a_/*K*_s_ online calculator tool [84].

### 4.13. Data Analysis

Data were analyzed with Excel 2003. The Student’s *t*-test was used to compare the differences. R 3.0.1 package [85], gplots [86], and ggplot2 [87] were used to draw the heatmap and other figures.

## 5. Conclusions

*Glyma04g39610* encodes *GmBRI1b*, which functions as a BR receptor. GmBRI1b is cell membrane protein. Ectopic over-expression of *GmBRI1b* in *bri1-6* and *bri1-5 bak1-1D* rescues the BR signaling-related growth and development defects of the two mutants. The *Glycine max* genome contains six BR receptor-encoding genes, which are grouped into three clades and are generated from three gene duplication events during evolution. BR receptors in plants were subjected to purifying selection during evolution.

## Figures and Tables

**Table 1:** General information about the brassinosteroid (BR) receptor genes in soybean based on bioinformatics analysis.

Gene	Locus	EST	TM	SP	KD	Length (AA)	Localization	Int/Ext
*GmBRI1a*	*Glyma06g15270*	Yes	784..806	1..20	871..1143	1184	Plas	0/1
*GmBRI1b*	*Glyma04g39610*	Yes	787..809	1..22	874..1146	1187	Plas	0/1
*GmBRL1a*	*Glyma04g12860*	Yes	827..849	1..43	928..1202	1207	Cyto	0/1
*GmBRL1b*	*Glyma06g47870*	Yes	843..865	No	912..1186	1211	Plas	0/1
*GmBRL2a*	*Glyma05g26771*	Yes	752..774	1..32	756..1038	1053	Nucl	1/2
*GmBRL2b*	*Glyma08g09750*	Yes	no	1..29	837..1121	1136	Plas	0/1

The transmembrane domains (TM) signal peptides (SP), and kinase domains (KD) were predicted by the SMART [44] program and the positions (from the amino terminus to the carboxyl terminus) of the TM, SP, and KD are indicated in the table. Putative cell localization of the soybean BR receptors was predicted by PSORT [45]. Based on the released genome sequences and cDNA sequences of soybean, the numbers of introns and exons were determined through SIM4 as described in Methods. AA, amino acid; Cyto, cytoplasm; EST, expressed sequence tag; Ext, Extron; Int, intron; Nucl, nucleus; Plas, plasmamembrane.

## Supplementary Materials

Supplementary materials can be found at http://www.mdpi.com/1422-0067/17/6/897/s1.

## Abbreviations

AA	Amino acid
BAK1	BRI1 ASSOCIATED RECEPTOR KINASE 1
BIN2	BRASSINOSTEROID INSENSITIVE2
BKI1	BRI1 KINASE INHIBITOR 1
BR	Brassinosteroid
BRI1	BRASSINOSTEROID INSENSITIVE 1
Brz	Brassinazole
BZR1	BRASSINAZOLE-RESISTANT1
ID	Island domain
ORF	Open reading frame
qRT-PCR	Quantitative real-time PCR

## References

[1] Müssig C., Shin G.H., Altmann T. (2003). Brassinosteroids promote root growth in *Arabidopsis*. Plant Physiol..

[2] Hacham Y., Holland N., Butterfield C., Ubeda-Tomas S., Bennett M.J., Chory J., Savaldi-Goldstein S. (2011). Brassinosteroid perception in the epidermis controls root meristem size. Development.

[3] Kim T.W., Michniewicz M., Bergmann D.C., Wang Z.Y. (2012). Brassinosteroid regulates stomatal development by GSK3-mediated inhibition of a MAPK pathway. Nature.

[4] Leubner-Metzger G. (2001). Brassinosteroids and gibberellins promote tobacco seed germination by distinct pathways. Planta.

[5] Chory J., Nagpal P., Peto C.A. (1991). Phenotypic and genetic analysis of *det2*, a new mutant that affects light-regulated seedling development in *Arabidopsis*. Plant Cell.

[6] Szekeres M., Németh K., Koncz-Kálmán Z., Mathur J., Kauschmann A., Altmann T., Rédei G.P., Nagy F., Schell J., Koncz C. (1996). Brassinosteroids rescue the deficiency of cyp90, a cytochrome p450, controlling cell elongation and de-etiolation in *Arabidopsis*. Cell.

[7] Bai M.Y., Fan M., Oh E., Wang Z.Y. (2012). A triple helix-loop-helix/basic helix-loop-helix cascade controls cell elongation downstream of multiple hormonal and environmental signaling pathways in *Arabidopsis*. Plant Cell.

[8] Ferguson B.J., Ross J.J., Reid J.B. (2005). Nodulation phenotypes of gibberellin and brassinosteroid mutants of pea. Plant Physiol..

[9] Nakashita H., Yasuda M., Nitta T., Asami T., Fujioka S., Arai Y., Sekimata K., Takatsuto S., Yamaguchi I., Yoshida S. (2003). Brassinosteroid functions in a broad range of disease resistance in tobacco and rice. Plant J..

[10] Dhaubhadel S., Browning K.S., Gallie D.R., Krishna P. (2002). Brassinosteroid functions to protect the translational machinery and heat-shock protein synthesis following thermal stress. Plant J..

[11] Bajguz A., Hayat S. (2009). Effects of brassinosteroids on the plant responses to environmental stresses. Plant Physiol. Biochem..

[12] Sathiyamoorthy P., Nakamuracohen S. (1990). *In vitro* root induction by 24-epibrassinolide on hypocotyl segments of soybean [*Glycine max* (l.) merr.]. Plant Growth Regul..

[13] Zurek D.M., Rayle D.L., McMorris T.C., Clouse S.D. (1994). Investigation of gene expression, growth kinetics, and wall extensibility during brassinosteroid-regulated stem elongation. Plant Physiol..

[14] Zhang M., Zhai Z., Tian X., Duan L., Li Z. (2008). Brassinolide alleviated the adverse effect of water deficits on photosynthesis and the antioxidant of soybean (*Glycine max* L.). Plant Growth Regul..

[15] Li J., Chory J. (1997). A putative leucine-rich repeat receptor kinase involved in brassinosteroid signal transduction. Cell.

[16] Wang Z.Y., Nakano T., Gendron J., He J., Chen M., Vafeados D., Yang Y., Fujioka S., Yoshida S., Asami T. (2002). Nuclear-localized BZR1 mediates brassinosteroid-induced growth and feedback suppression of brassinosteroid biosynthesis. Dev. Cell.

[17] Wang X., Chory J. (2006). Brassinosteroids regulate dissociation of BKI1, a negative regulator of BRI1 signaling, from the plasma membrane. Science.

[18] Li J., Wen J., Lease K.A., Doke J.T., Tax F.E., Walker J.C. (2002). BAK1, an *Arabidopsis* LRR receptor-like protein kinase, interacts with BRI1 and modulates brassinosteroid signaling. Cell.

[19] Li J., Nam K.H., Vafeados D., Chory J. (2001). *BIN2*, a new brassinosteroid-insensitive locus in *Arabidopsis*. Plant Physiol..

[20] Li J., Nam K.H. (2002). Regulation of brassinosteroid signaling by a GSK3/SHAGGY-like kinase. Science.

[21] He J.X., Gendron J.M., Yang Y., Li J., Wang Z.Y. (2002). The GSK3-like kinase BIN2 phosphorylates and destabilizes BZR1, a positive regulator of the brassinosteroid signaling pathway in *Arabidopsis*. Proc. Natl. Acad. Sci. USA.

[22] Zhao J., Peng P., Schmitz R.J., Decker A.D., Tax F.E., Li J. (2002). Two putative BIN2 substrates are nuclear components of brassinosteroid signaling. Plant Physiol..

[23] Tang W., Yuan M., Wang R., Yang Y., Wang C., Oses-Prieto J.A., Kim T.W., Zhou H.W., Deng Z., Gampala S.S. (2001). PP2A activates brassinosteroid-responsive gene expression and plant growth by dephosphorylating BZR1. Nat. Cell Biol..

[24] Yin Y., Vafeados D., Tao Y., Yoshida S., Asami T., Chory J. (2005). A new class of transcription factors mediates brassinosteroid-regulated gene expression in *Arabidopsis*. Cell.

[25] Peng P., Yan Z., Zhu Y., Li J. (2008). Regulation of the *Arabidopsis* GSK3-like kinase BRASSINOSTEROID-INSENSITIVE 2 through proteasome-mediated protein degradation. Mol. Plant.

[26] Wang X., Li X., Meisenhelder J., Hunter T., Yoshida S., Asami T., Chory J. (2005). Autoregulation and homodimerization are involved in the activation of the plant steroid receptor BRI1. Dev. Cell.

[27] Hothorn M., Belkhadir Y., Dreux M., Dabi T., Noel J.P., Wilson I.A., Chory J. (2011). Structural basis of steroidhormone perception by the receptor kinase BRI1. Nature.

[28] She J., Han Z., Kim T.W., Wang J., Cheng W., Chang J., Shi S., Wang J., Yang M., Wang Z.Y. (2011). Structural insight into brassinosteroid perception by BRI1. Nature.

[29] Cano-Delgado A., Yin Y., Yu C., Vafeados D., Mora-Garcia S., Cheng J.C., Nam K.H., Li J., Chory J. (2004). BRL1 and BRL3 are novel brassinosteroid receptors that function in vascular differentiation in *Arabidopsis*. Development.

[30] Zhou A., Wang H., Walker J.C., Li J. (2004). BRL1, a leucine-rich repeat receptor-like protein kinase, is functionally redundant with BRI1 in regulating *Arabidopsis* brassinosteroid signaling. Plant J..

[31] Yamamuro C., Ihara Y., Wu X., Noguchi T., Fujioka S., Takatsuto S., Ashikari M., Kitano H., Matsuoka M. (2000). Loss of function of a rice *brassinosteroid insensitive1* homolog prevents internode elongation and bending of the lamina joint. Plant Cell.

[32] Nakamura A., Fujioka S., Sunohara H., Kamiya N., Hong Z., Inukai Y., Miura K., Takatsuto S., Yoshida S., Ueguchi-Tanaka M. (2006). The role of *OsBRI1* and its homologous genes, *OsBRL1* and *OsBRL3*, in rice. Plant Physiol..

[33] Montoya T., Nomura T., Farrar K., Kaneta T., Yokota T., Bishop G.J. (2002). Cloning the tomato *Curl3* gene highlights the putative dual role of the leucine-rich repeat receptor kinase tBRI1/SR160 in plant steroid hormone and peptide hormone signaling. Plant Cell.

[34] Nomura T., Bishop G.J., Kaneta T., Reid J.B., Chory J., Yokota T. (2003). The *LKA* gene is *BRASSINOSTEROID INSENSITIVE 1* homolog of pea. Plant J..

[35] Chono M., Honda I., Zeniya H., Yoneyama K., Saisho D., Takeda K., Takatsuto S., Hoshino T., Watanabe Y. (2003). A semidwarf phenotype of barley uzu results from a nucleotide substitution in the gene encoding a putative brassinosteroid receptor. Plant Physiol..

[36] Sun Y., Fokar M., Asami T., Yoshida S., Allen R.D. (2004). Characterization of the brassinosteroid insensitive 1genes of cotton. Plant Mol. Biol..

[37] Kir G., Ye H., Nelissen H., Neelakandan A.K., Kusnandar A.S., Luo A., Inzé D., Sylvester A.W., Yin Y., Becraft P.W. (2015). RNA interference knockdown of BRASSINOSTEROID INSENSITIVE1 in maize reveals novel functions for brassinosteroid signaling in controlling plant architecture. Plant Physiol..

[38] Navarro C., Moore J., Ott A., Baumert E., Mohan A., Gill K.S., Sandhu D. (2015). Evolutionary, comparative and functional analyses of the brassinosteroid receptor gene, BRI1, in wheat and its relation to other plant genomes. PLoS ONE.

[39] Wang M., Sun S., Wu C., Han T., Wang Q. (2014). Isolation and characterization of the brassinosteroid receptor gene (*GmBRI1*) from *Glycine max*. Int. J. Mol. Sci..

[40] Schmutz J., Cannon S., Schlueter J., Ma J., Mitros T. (2010). Genome sequence of the palaeopolyploid soybean. Nature.

[41] Childs K.L., Hamilton J.P., Zhu W., Ly E., Cheung F., Wu H., Rabinowicz P.D., Town C.D., Buell C.R., Chan A.P. (2007). The TIGR plant transcript assemblies database. Nucleic Acids Res..

[42] Wheeler D.L., Chappey C., Lash A.E., Leipe D.D., Madden T.L., Schuler G.D., Tatusova T.A., Rapp B.A. (2000). Database resources of the National Center for Biotechnology Information. Nucleic Acids Res..

[43] Goodstein D.M., Shu S., Howson R., Neupane R., Hayes R.D., Fazo J., Mitros T., Dirks W., Hellsten U., Putnam N. (2012). Phytozome: A comparative platform for green plant genomics. Nucleic Acids Res..

[44] Letunic I., Doerks T., Bork P. (2015). SMART: Recent updates, new developments and status. Nucleic Acids Res..

[45] Horton P., Park K.J., Obayashi T., Fujita N., Harada H., Adams-Collier C.J., Nakai K. (2007). WoLF PSORT: Protein localization predictor. Nucleic Acids Res..

[46] Lee T.H., Tang H., Wang X., Paterson A.H. (2013). PGDD: A database of gene and genome duplication in plants. Nucleic Acids Res..

[47] Severin A.J., Woody J.L., Bolon Y.T., Joseph B., Diers B.W., Farmer A.D., Muehlbauer G.J., Nelson R.T., Grant D., Specht J.E. (2010). RNA-Seq atlas of *Glycine max*: A guide to the soybean transcriptome. BMC Plant Biol..

[48] Nelson B.K., Cai X., Nebenführ A. (2007). A multicolored set of in vivo organelle markers for co-localization studies in Arabidopsis and other plants. Plant J..

[49] Noguchi T., Fujioka S., Choe S., Takatsuto S., Yoshida S., Yuan H., Feldmann K.A., Tax F.E. (1999). Brassinosteroid-insensitive dwarf mutants of *Arabidopsis* accumulate brassinosteroids. Plant Physiol..

[50] Li J., Jin H. (2007). Regulation of brassinosteroid signaling. Trends Plant Sci..

[51] Nagata N., Min Y.K., Nakano T., Asami T., Yoshida S. (2000). Treatment of dark-grown *Arabidopsis thaliana* with a brassinosteroid-biosynthesis inhibitor, brassinazole, induces some characteristics of light-grown plants. Planta.

[52] Wang X., Kota U., He K., Blackburn K., Li J., Goshe M.B., Huber S.C., Clouse S.D. (2008). Sequential transphosphorylation of the BRI1/BAK1 receptor kinase complex impacts early events in brassinosteroid signaling. Dev. Cell.

[53] Mathur J., Molnár G., Fujioka S., Takatsuto S., Sakurai A., Yokota T., Adam G., Voigt B., Nagy F., Maas C. (1998). Transcription of the *Arabidopsis CPD* gene, encoding a steroidogenic cytochrome p450, is negatively controlled by brassinosteroids. Plant J..

[54] Bancos S., Nomura T., Sato T., Molnár G., Bishop G.J., Koncz C., Yokota T., Nagy F., Szekeres M. (2002). Regulation of transcript levels of the *Arabidopsis* cytochrome p450 genes involved in brassinosteroid biosynthesis. Plant Physiol..

[55] Tanaka K., Asami T., Yoshida S., Nakamura Y., Matsuo T., Okamoto S. (2005). Brassinosteroid homeostasis in *Arabidopsis* is ensured by feedback expressions of multiple genes involved in its metabolism. Plant Physiol..

[56] Pieper U., Webb B.M., Barkan D.T., Schneidman-Duhovny D., Schlessinger A., Braberg H., Yang Z., Meng E.C., Pettersen E.F., Huang C.C. (2011). ModBase, a database of annotated comparative protein structure models, and associated resources. Nucleic Acids Res..

[57] Berman H.M., Westbrook J., Feng Z., Gilliland G., Bhat T.N., Weissig H., Shindyalov I.N., Bourne P.E. (2000). The Protein Data Bank. Nucleic Acids Res..

[58] Guindon S., Dufayard J.F., Lefort V., Anisimova M., Hordijk W., Gascuel O. (2010). New algorithms and methods to estimate maximum-likelihood phylogenies: Assessing the performance of PhyML3.0. Syst. Biol..

[59] Fiser A., Do R.K., Sali A. (2000). Modeling of loops in protein structures. Protein Sci..

[60] PyMOL The PyMOL molecular graphics system, version 1.5.0. https://www.pymol.org.

[61] Huelsenbeck J.P., Ronquist F. (2001). Mrbayes: Bayesian inference of phylogenetic trees. Bioinformatics.

[62] Suyama M., Torrents D., Bork P. (2006). PAL2NAL: Robust conversion of protein sequence alignments into the corresponding codon alignments. Nucleic Acids Res..

[63] Kutschera U., Wang Z.Y. (2012). Brassinosteroid action in flowering plants: A Darwinian perspective. J. Exp. Bot..

[64] Hayat S., Ahmad A. (2011). Brassinosteroids: A Class of Plant Hormone.

[65] Friedrichsen D.M., Joazeiro C.A., Li J., Hunter T., Chory J. (2000). Brassinosteroid-insensitive-1 is a ubiquitously expressed leucine-rich repeat receptor serine/threonine kinase. Plant Physiol..

[66] Terakado J., Fujihara S., Goto S., Kuratani R., Suzuki Y., Yoshida S., Yoneyama T. (2005). Systemic effect of a brassinosteroid on root nodule formation in soybean as revealed by the application of brassinolide and brassinazole. Soil Sci. Plant Nutr..

[67] Libault M., Joshi T., Takahashi K., Hurley-Sommer A., Puricelli K., Blake S., Finger R.E., Taylor C.G., Xu D., Nguyen H.T. (2009). Large-scale analysis of putative soybean regulatory gene expression identifies a *Myb* gene involved in soybean nodule development. Plant Physiol..

[68] Li J., Nagpal P., Vitart V., McMorris T.C., Chory J. (1996). A role for brassinosteroids in light-dependent development of *Arabidopsis*. Science.

[69] Choe S., Dilkes B.P., Fujioka S., Takatsuto S., Sakurai A., Feldmann K.A. (1998). The *DWF4* gene of *Arabidopsis* encodes a cytochrome p450 that mediates multiple 22α-hydroxylation steps in brassinosteroid biosynthesis. Plant Cell.

[70] Bishop G.J., Harrison K., Jones J.D. (1996). The tomato *Dwarf* gene isolated by heterologous transposon tagging encodes the first member of a new cytochrome p450 family. Plant Cell.

[71] Wang H., Mao H. (2014). On the origin and evolution of plant brassinosteroid receptor kinases. J. Mol. Evol..

[72] Cheon J., Fujioka S., Dilkes B.P., Choe S. (2013). Brassinosteroids regulate plant growth through distinct signaling pathways in *Selaginella* and *Arabidopsis*. PLoS ONE.

[73] Singh A.P., Pandey B.K., Deveshwar P., Narnoliya L., Parida S.K., Giri J. (2015). JAZ repressors: Potential involvement in nutrients deficiency response in rice and chickpea. Front. Plant Sci..

[74] Zhang S.D., Ling L.Z., Yi T.S. (2015). Evolution and divergence of SBP-box genes in land plants. BMC Genom..

[75] Victoria F.C., Bervald C.M., daMaia L.C., deSousa R.O., Panaud O., deOliveira A.C. (2012). Phylogenetic relationships and selective pressure on gene families related to iron homeostasis in land plants. Genome.

[76] Marshall O.J. (2004). PerlPrimer: Cross-platform, graphical primer design for standard, bisulphite and real-time PCR. Bioinformatics.

[77] Schmittgen T.D., Zakrajsek B.A., Mills A.G., Gorn V., Singer M.J., Reed M.W. (2000). Quantitative reverse transcription-polymerase chain reaction to study mRNA decay: Comparison of endpoint and real-time methods. Anal. Biochem..

[78] Clough S.J., Bent A.F. (1998). Floral dip: A simplified method for Agrobacterium-mediated transformation of *Arabidopsis thaliana*. Plant J..

[79] Liu T.Y., Huang T.K., Tseng C.Y., Lai Y.S., Lin S.I., Lin W.Y., Chen J.W., Chiou T.J. (2012). PHO2-dependent degradation of PHO1 modulates phosphate homeostasis in *Arabidopsis*. Plant Cell.

[80] Florea L., Hartzell G., Zhang Z., Rubin G. M., Miller W. (1998). A computer program for aligning a cDNA sequence with a genomic DNA sequence. Genome Res..

[81] Notredame C., Higgins D.G., Heringa J. (2000). T-coffee: A novel method for fast and accurate multiple sequence alignment. J. Mol. Biol..

[82] Sali A., Blundell T.L. (1993). Comparative protein modelling by satisfaction of spatial restraints. J. Mol. Biol..

[83] Shen M.Y., Sali A. (2006). Statistical potential for assessment and prediction of protein structures. Protein Sci..

[84] Liberles D.A. (2001). Evaluation of methods for determination of a reconstructed history of gene sequence evolution. Mol. Biol. Evol..

[85] R Core Team R: A Language and Environment for Statistical Computing. http://www.R-project.org/.

[86] Warnes G.R., Bolker B., Bonebakker L., Gentleman R., Huber W., Liaw W., Lumley T., Maechler M., Magnusson A., Moeller S. gplots: Various R programming tools for plotting data. http://CRAN.R-project.org/package=gplots.

[87] Wickham H. (2009). ggplot2: Elegant Graphics for Data Analysis.

