# Supplementary material for: Functional Characterization of Soybean Glyma04g39610 as a Brassinosteroid Receptor Gene and Evolutionary Analysis of Soybean Brassinosteroid Receptors
Source: Int J Mol Sci. 2016 Jun 7;17(6):897. doi: 10.3390/ijms17060897 (PMC4926431; doi:10.3390/ijms17060897)
Supplement: Supplementary file 1 [file ijms-17-00897-s001.zip › ijms-128646-Supplementary Materials/ijms-128646-supplementary.pdf]

SupplementaryMaterials: Functional Characterization of Soybean *Glyma04g39610* as a Brassinosteroid Receptor Gene and Evolutionary Analysis of Soybean Brassinosteroid Receptors

Suna Peng, Ping Tao, Feng Xu, Aiping Wu, Weige Huo and Jinxiang Wang

Table S1. Sequence finger prints of the LLR domains in GmBRI1b. The cysteine residues in position 1 to 24 are shown in blue.

LRR	*beg*	1 L	2 X	3 X	4 L	5 X	6 X	7 L	8 X	9 L	10 S	11 X	12 N	13 X	14 L	15 S	16 G	17 X	18 I	19 P	20 X	21 X	22 L	23 G	24 X	*end*	No. res
**1**	*47*	L	T	S	I	D	L	S	S	V	P	L	S	T	N	L	T	V	I	A	S	F	L	L	S	*70*	24
**2**	*71*	L	D	H	L	Q	S	L	S	LK	S	T	N	L	S	G	P	A	A	M	P	P	L	S	H	*95*	25
**3**	*99*	S	S	S	L	T	S	L	D	L	S	Q	N	S	L	S	A	S	L	N	D	M	S	FL	AS	*124*	26
**4**	*125*	C	S	N	L	Q	S	L	N	L	S	S	N	L	L	Q	F	G		P	P	P	H	W	K	*146*	22
**5**	*148*	L	H	H	L	R	F	A	D	F	S	Y	N	K	I	S	G	P	G	V	V	S	WL	LN	P	*173*	26
**6**	*174*	V	I	E	L	L	S	L	K	G	N	K	V	T	G	E	T	D					F	S	G	*193*	20
**7**	*194*	S	I	S	L	Q	Y	L	D	L	S	S	N	N	F	S	V	T	L	P	T		F	G	E	*216*	23
**8**	*217*	C	S	S	L	E	Y	L	D	L	S	A	N	K	Y	L	G	D	I	A	R	T	L	S	P	*240*	24
**9**	*241*	C	K	S	L	V	Y	L	N	V	S	S	N	Q	F	S	G	P	V	P			S	L	P	*262*	22
**10**	*263*	S	G	S	L	Q	F	V	Y	L	A	A	N	H	F	H	G	Q	I	P	L	S	L	AD	L	*287*	25
**11**	*288*	C	S	T	L	L	Q	L	D	L	S	S	N	N	L	T	G	A	L	P	G	A	F	G	A	*311*	24
**12**	*312*	C	T	S	L	Q	S	L	D	I	S	S	N	L	F	A	G	A	L	P	M	SV	L	T	Q	*336*	25
**13**	*337*	M	T	S	L	K	E	L	A	V	A	F	N	G	F	L	G	A	L	P	E	S	L	S	K	*360*	24
**14**	*361*	L	S	A	L	E	L	L	D	L	S	S	N	N	F	S	G	S	I	P	A	S	L	C		*383*	23
**15**	*392*		N	N	L	K	E	L	Y	L	Q	N	N	R	F	T	G	F	I	P	P	T	L	S	N	*414*	22
**16**	*415*	C	S	N	L	V	A	L	D	L	S	F	N	F	L	T	G	T	I	P	P	S	L	G	S	*438*	24
**17**	*439*	L	S	N	L	K	D	F	I	I	W	L	N	Q	L	H	G	E	I	P	Q	E	L	M	Y	*462*	24
**18**	*463*	L	K	S	L	E	N	L	I	L	D	F	N	D	L	T	G	N	I	P	S	G	L	V	N	*486*	24
**19**	*487*	C	T	K	L	N	W	I	S	L	S	N	N	R	L	S	G	E	I	P	P	W	I	G	K	*510*	24
**20**	*511*	L	S	N	L	A	I	L	K	L	S	N	N	S	F	S	G	R	I	P	P	E	L	G	D	*534*	24
**21**	*535*	C	T	S	L	I	W	L	D	L	N	T	N	M	L	T	G	P	I	P	P	E	LF	K	Q	*559*	25
**22**	*629*			S	M	I	F	L	D	I	S	H	N	M	L	S	G	S	I	P	K	E	I	G	A	*650*	22
**23**	*651*	M	Y	Y	L	Y	I	L	N	L	G	H	N	N	V	S	G	S	I	P	Q	E	L	G	K	*674*	24
**24**	*675*	M	K	N	L	N	I	L	D	L	S	N	N	R	L	E	G	Q	I	P	Q	S	L	T	G	*698*	24
**25**	*699*	L	S	L	L	T	E	I	D	L	S	N	N	L	L	T	G	T	I	P	E	S		G	Q	*721*	23

Table S2. List of the quality criteria parameters in automatic modeling with modeller v9.10 at ModWeb (http://modbase.compbio.ucsf.edu/ModWeb20-html/modweb.html) [56].

Gene Name	Target Region	Protein Length	Template PDB Code	Template Region	Sequence Identity	E-Value	GA341	MPQS	z-DOPE	TSVMod Method	TSVMod RMSD	TSVMod NO35
GmBRI1a	21–761	762	3RGZ	30–770	60.00%	0	1.00	1.73604	−0.97	MSRED	2.024	0.84
GmBRI1b	3–744	745	3RGZ	30–770	60.00%	0	1.0	1.75347	−0.93	MSALL	1.006	0.913
GmBRL1a	68–824	825	3RGX	34–770	41.0%	0	1.00	1.39568	−0.38	MSRED	8.004	0.612
GmBRL1b	65–808	809	3RGX	50–770	44.00%	0	1.0	1.41525	−0.45	MSRED	2.051	0.8
GmBRL2a	34–724	725	3RGX	31–770	43.0%	0	1.0	1.4216	−0.49	MSRED	4.557	0.707
GmBRL2b	35–725	726	3RGX	31–770	43.00%	0	1.0	1.41229	−0.43	MSRED	5.131	0.645

Table S3. List of primers used in this study.

Primer Name	Sequence (5′-3′)	Usage
GmBRI1b.F	ATGAAAGCTCTGTACTACAGAAGCT	Full *GmBRI1b* cloning
GmBRI1b.R	ATGCTTGCTCAATTCAGGGGC	Full *GmBRI1b* cloning
GmBRI1b.F1	GATGGCAATGTTCAAGGAGATTCAGGCG	sequencing
GmBRI1b.F2	CGAGTGTTCTTCGCTTGAGT	sequencing
GmBRI1b.R1	AATTCCCTGTCCCCTTGTCCGCTAA	sequencing
GmBRI1b.R2	CACATCCCCAAACCCGCCAGAGCCA	sequencing
GmEF1α.F	CTGGAGGTTTTGAGGCTGGTAT	qRT-PCR
GmEF1α.R	CCAAGGGTGAAAGCAAGAAGA	qRT-PCR
qGmBRI1b.F	TTGAAATGGTGGAGATGAGCA	qRT-PCR
qGmBRI1b.R	AGGGGAGCTGAATCCGTCA	qRT-PCR
qCPD.F	TTGCTCAACTCAAGGAAGAG	qRT-PCR
qCPD.R	TGATGTTAGCCACTCGTAGC	qRT-PCR
qDWF4.F	CATAAAGCTCTTCAGTCACGA	qRT-PCR
qDWF4.R	CGTCTGTTCTTTGTTTCCTAA	qRT-PCR
qBR6ox1.F	TCCCGTATCGGAGTCTTTGGT	qRT-PCR
qBR6ox1.R	TGGCCAATCTTTGGCGAA	qRT-PCR
qBR6ox2.F	CAATAGTCTCAATGGACGCAGAGT	qRT-PCR
qBR6ox2.R	AACCGCAGCTATGTTGCATG	qRT-PCR
qAtEF1a.F	GTCGATTCTGGAAAGTCGAC	qRT-PCR
qAtEF1a.R	AATGTCAATGGTGATACCACGC	qRT-PCR
GmBRI1bGFP.f	ATCGTCGACGATGAAAGCTCTGTACTACAGAAGCT	Subcellular localization
GmBRI1b.GFP.r	AAGCCCGGGCATGCTTGCTCAATTCAGGGGC	Subcellular localization
OX-GmBRI1b.F	ATCGTCGACATGAAAGCTCTGTACTACAGAAGCT	overexpression
OX-GMBRI1b.R	AAGCCCGGGATGCTTGCTCAATTCAGGGGC	Overexpression
GmBRI1b.RACE1	AATTCCCTGTCCCCTTGTCCGCTAA	5′RACE
GmBRI1b.RACE2	CACATCCCCAAACCCGCCAGAGCCA	5′RACE
GmBRI1b.RACE3	GATGGCAATGTTCAAGGAGATTCAGGCG	3′RACE
GmBRI1b.RACE4	CGAGTGTTCTTCGCTTGAGT	3′RACE
GmBRI1a.qF	TCCTCCTCATTAACCTCCCT	qRT-PCR
GmBRI1a.qR	TTGAGAAGCCAAGAAACGAC	qRT-PCR
GmBRI1b.qF	AACCTCCAATCCCTCAACCTC	qRT-PCR
GmBRI1b.qR	TTTGTTGCCTTTGAGAGAGA	qRT-PCR
GmBRL1a.qF	GTATGATCTACCTTGACCTTTCC	qRT-PCR
GmBRL1a.qR	AAGAAAGACCCTCCAATGCC	qRT-PCR
GmBRL1b.qF	AACAAACTAACGGGTCAACTCAG	qRT-PCR
GmBRL1b.qR	GAACTAGATTCTTGCACGAACC	qRT-PCR
GmBRL2a.qF	AATGACAACAGCCAAACCAC	qRT-PCR
GmBRI2a.qR	AAACCACCACACCCAATGAG	qRT-PCR
GmBRL2b.qF	CAAGAATGATAACAGCCAACCC	qRT-PCR
GmBRI2b.qR	AGCAACAGAGATGAGAATTCCC	qRT-PCR

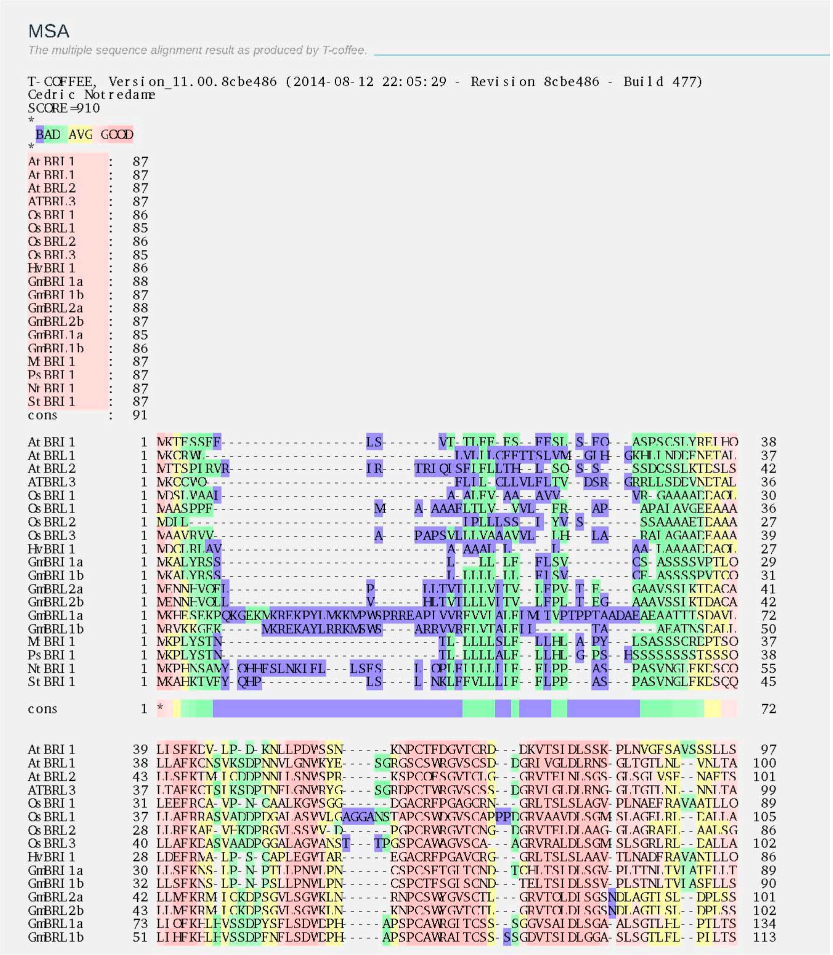

Figure S1. *Cont.*

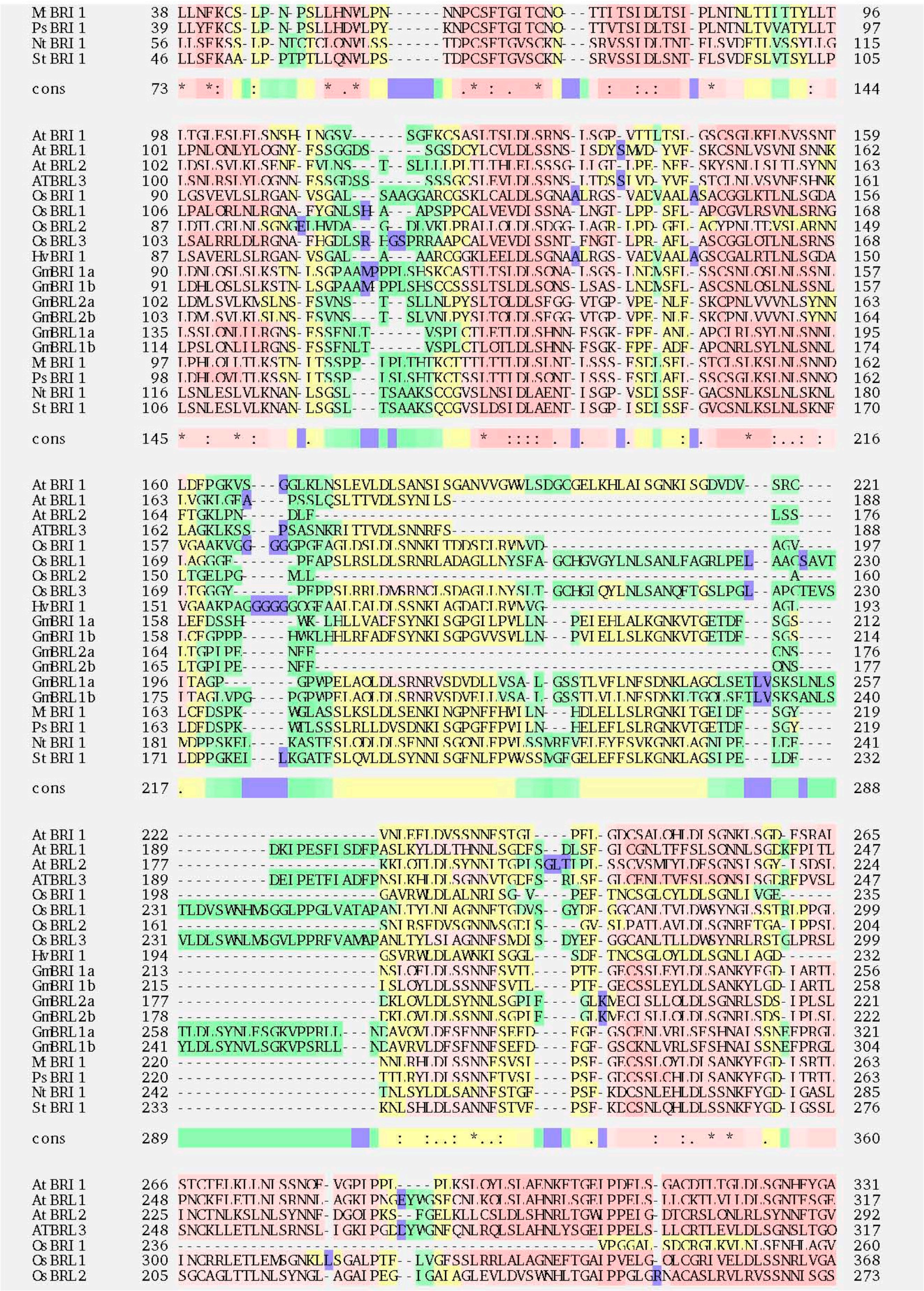

Figure S1. *Cont.*

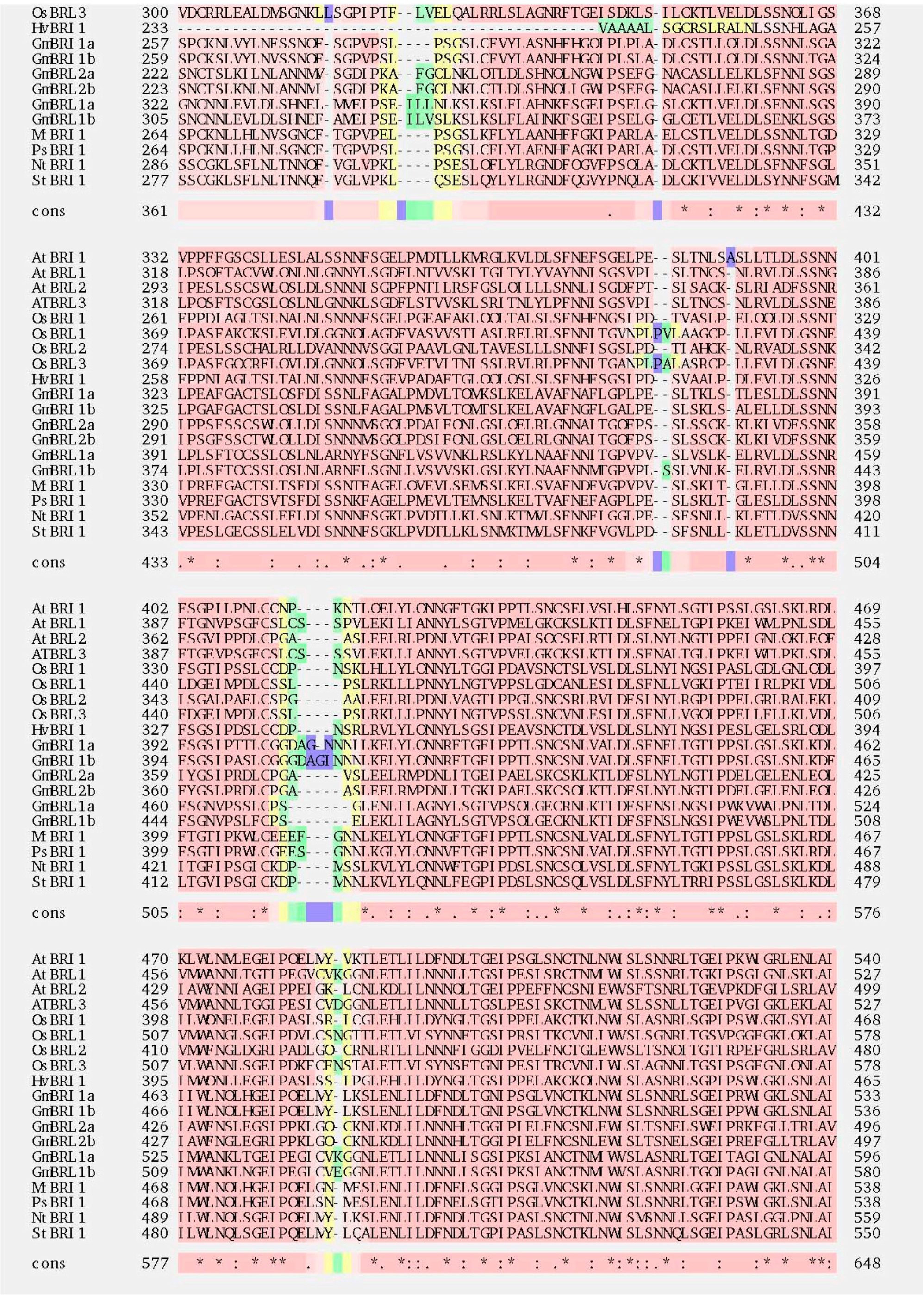

Figure S1. *Cont.*

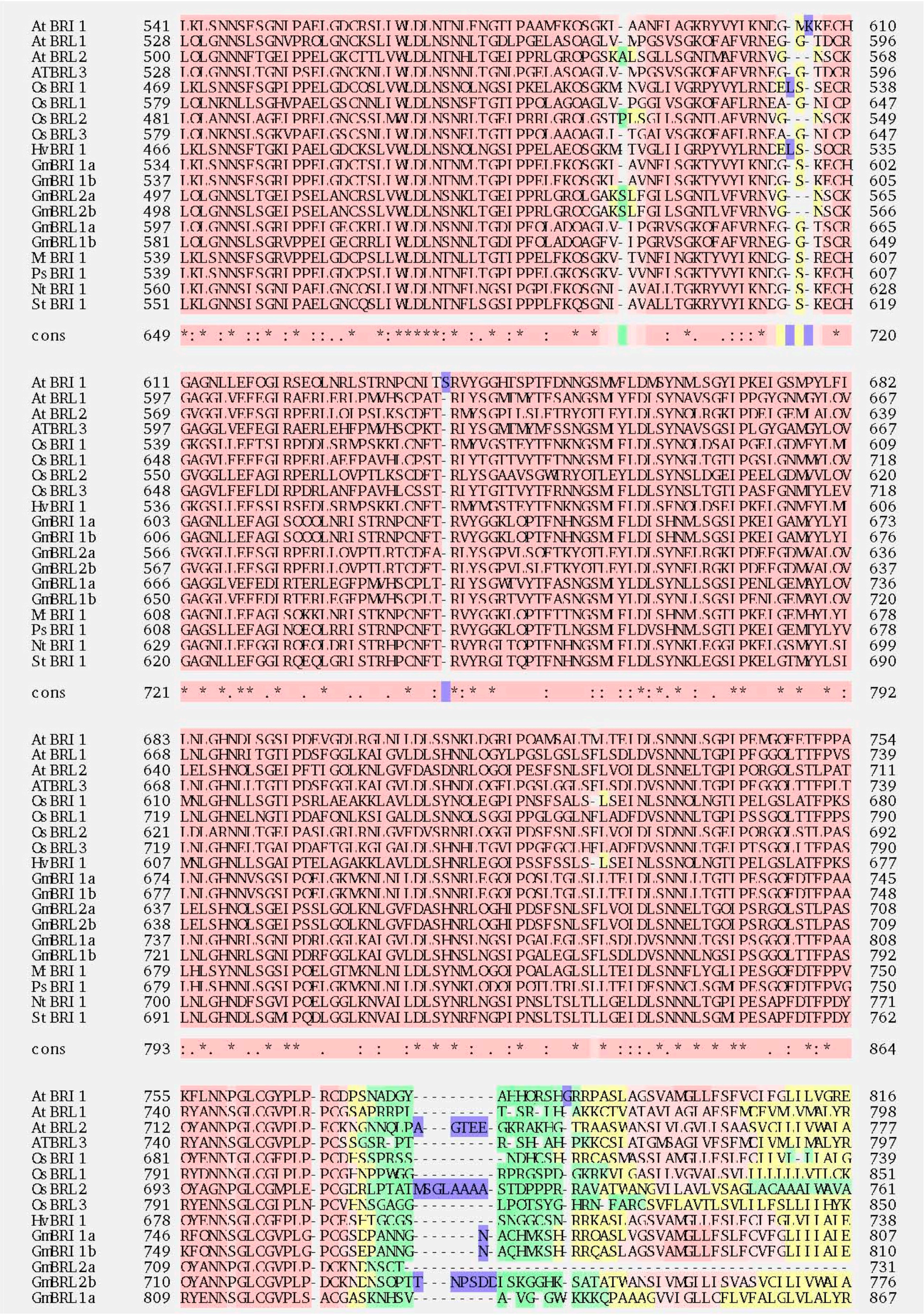

Figure S1. *Cont.*

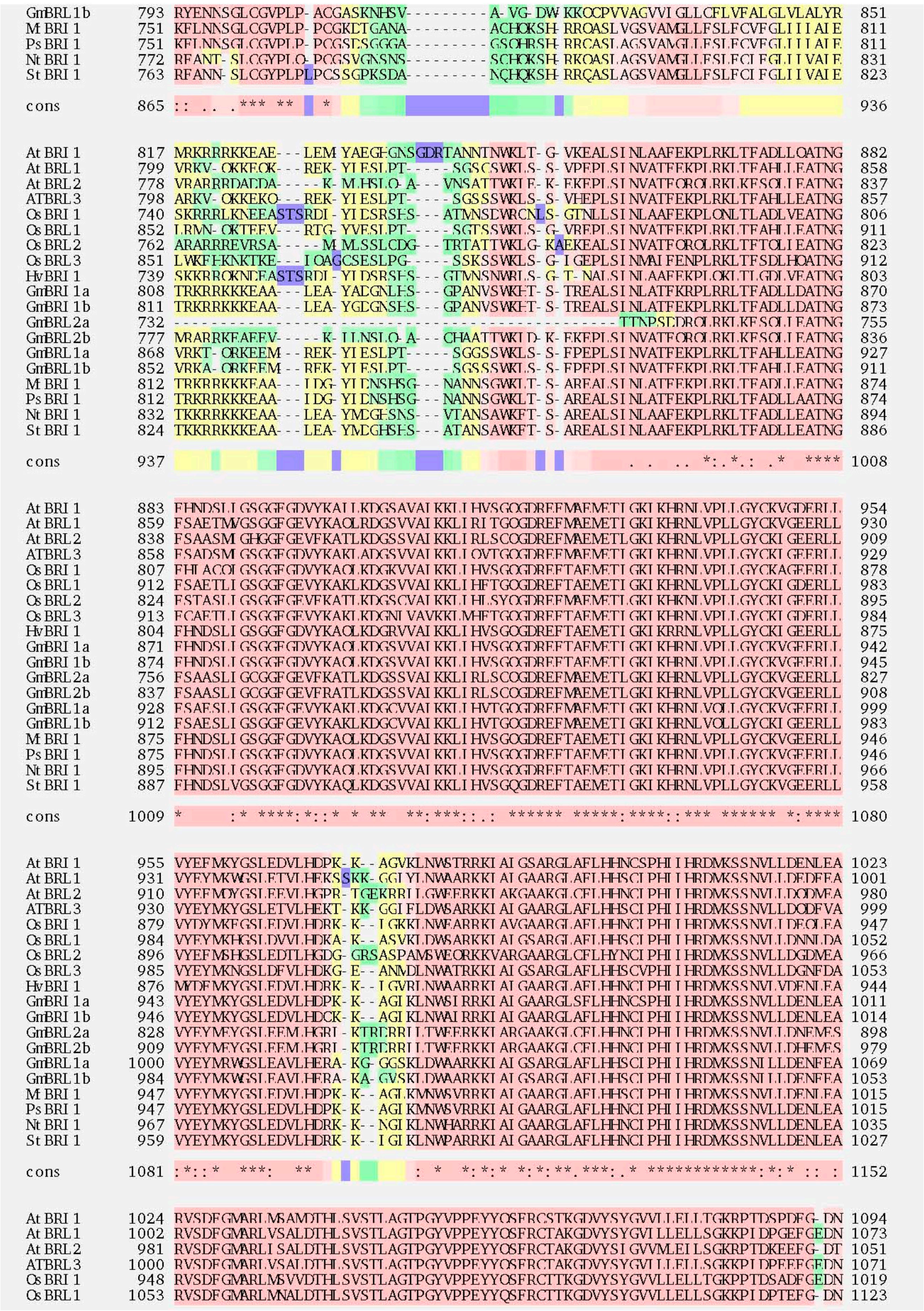

Figure S1. *Cont.*

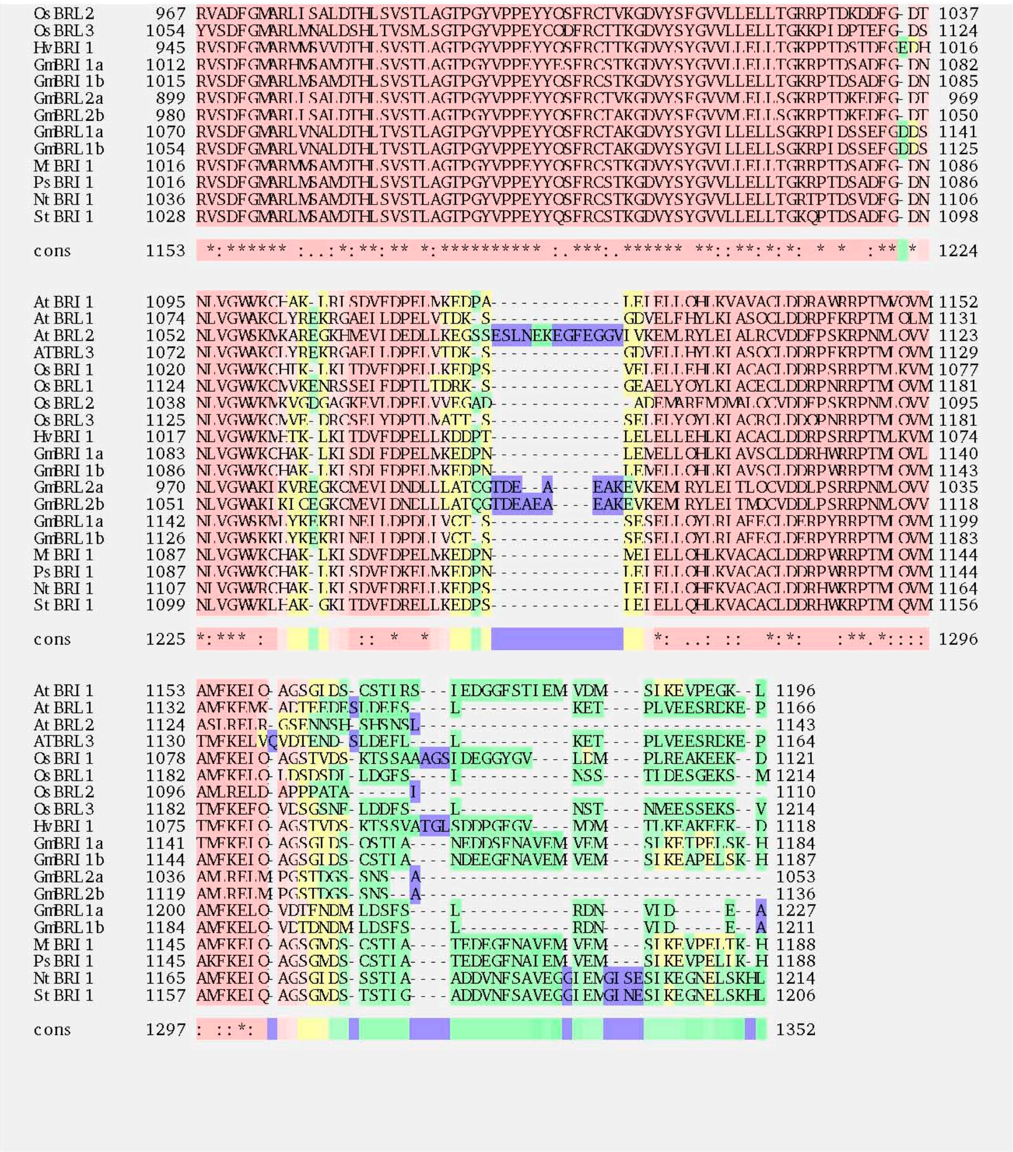

Figure S1. Alignment of the full amino acid sequences of the BR receptors from *Arabidopsis thaliana* (At), *Oryza sativa* (Os), *Glycine max* (Gm), *Solanum tuberosum* (St), *Medicago truncatula* (Mt), *Nicotiana tabacum* (Nt), and *Pisum sativum* (Ps). T-COFFEE V11.0 was used to construct the alignments [81]. The amino acid sequences that are identical in all aligned BR receptors are indicated with an asterisk (*), sequences that are conserved are indicated with a dot (. and :).

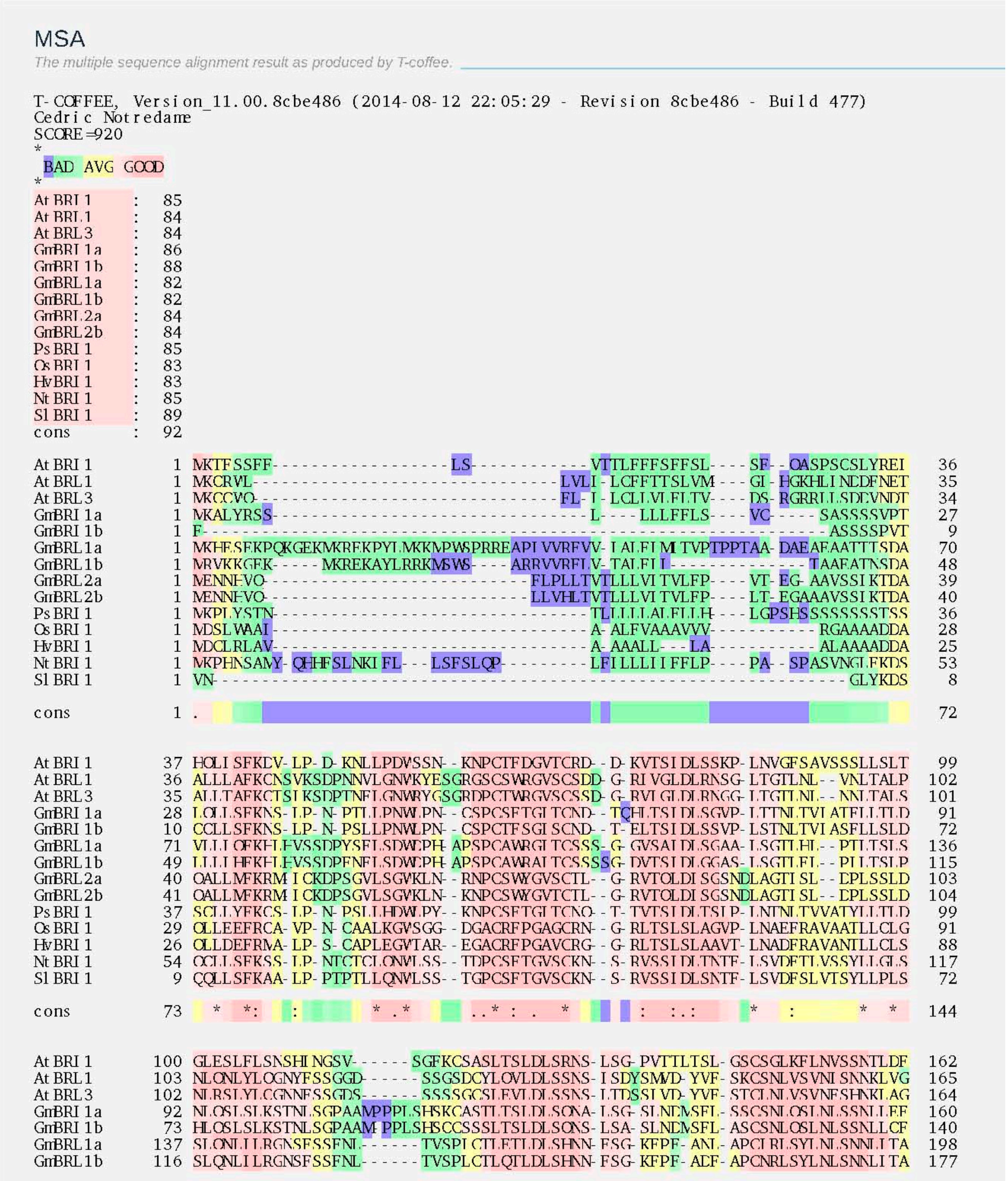

Figure S2. *Cont.*

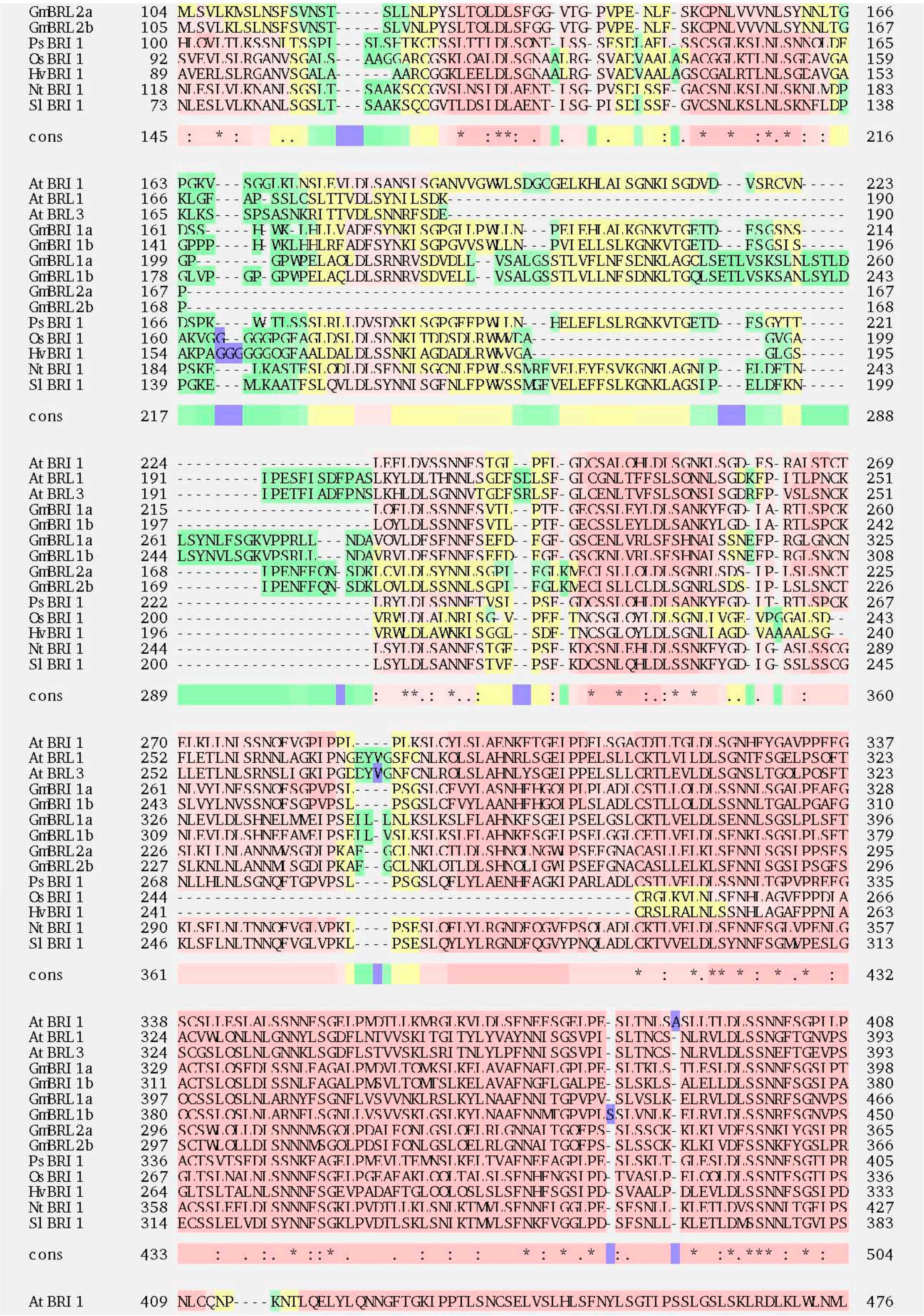

Figure S2. *Cont.*

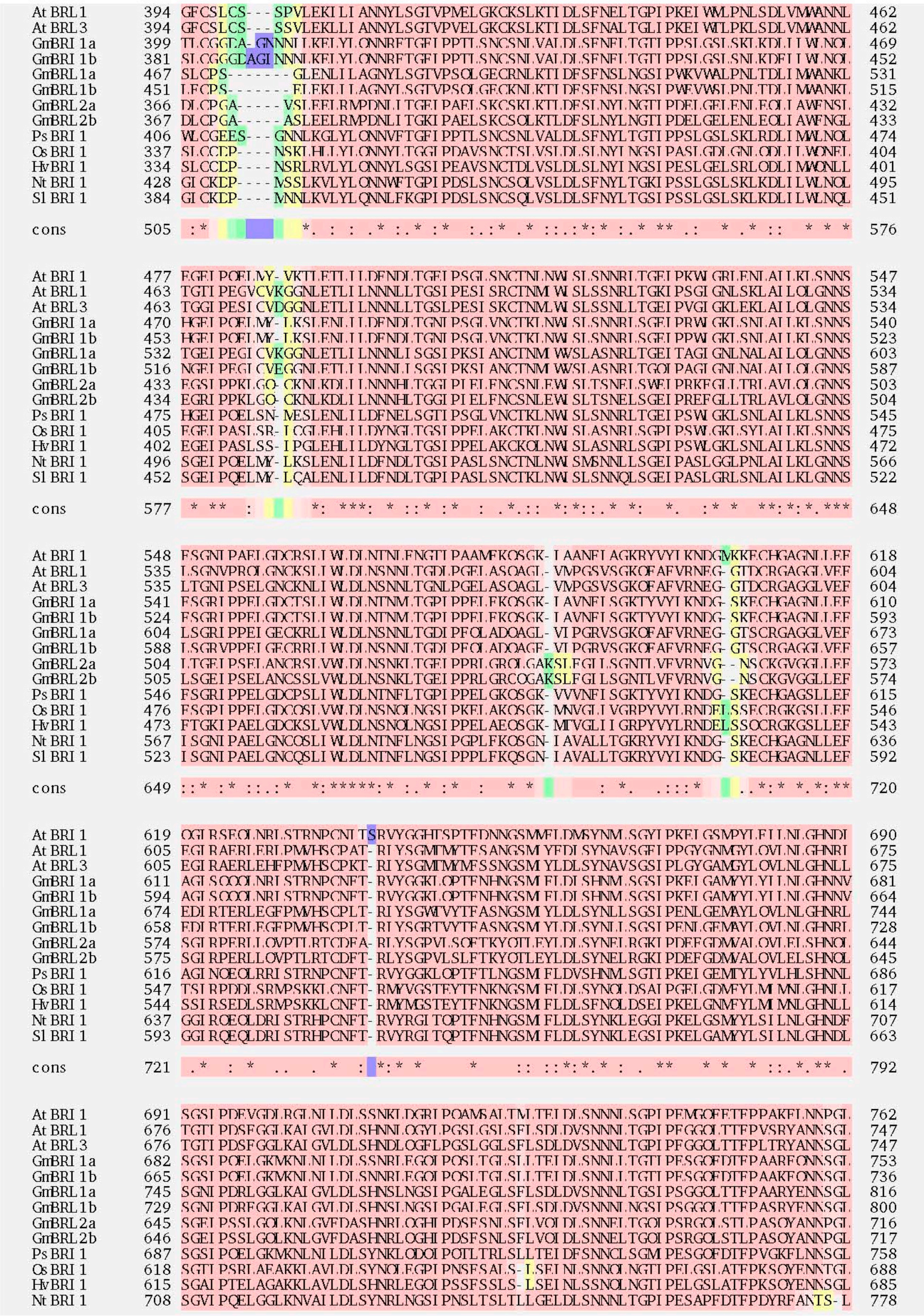

Figure S2. Alignment of the ectodomains of the BR receptors from Arabidopsis thaliana (At), Oryza sativa (Os), Glycine max (Gm), Solanum lycopersicum (Sl), Nicotiana tabacum (Nt), Pisum sativum (Ps), and Hordeum vulgare (Hv). T-COFFEE V11.0 was used to construct the alignments [81]. The amino acid sequences that are identical in all aligned BR receptors are indicated with an asterisk (*), sequences that are conserved are indicated with a dot (. and :).

```
SCORE=953
*
 BAD AVG GOOD
*
At BRI1     :  95
At BRL1     :  96
At BRL3     :  96
GmBRI1a     :  96
GmBRI1b     :  96
GmBRL1a     :  96
GmBRL1b     :  96
GmBRL2a     :  91
GmBRL2b     :  91
Ps BRI1     :  96
Os BRI1     :  95
HvBRI1      :  95
Nt BRI1     :  96
Sl BRI1     :  96
cons        :  95

At BRI1     1 SGK-IAANFLAGKRYVYIKNDGMKKECHGAGNLLEFOGIRSEOLNRLSTRNPCNITSRVYGGHTSPTFDNNG 71
At BRL1     1 AGL-VMPGSVSGKOFAFVRNEG-GTDCRGAGGLVEFEGIRAERLERLPMVHSCPAT-RIYSGMTMYTFSANG 69
At BRL3     1 AGL-VMPGSVSGKOFAFVRNEG-GTDCRGAGGLVEFEGIRAERLEHFPMVHSCPKT-RIYSGMTMYMFSSNG 69
GmBRI1a     1 SGK-IAVNFLSGKTYVYIKNDG-SKECHGAGNLLEFAGISOOOLNRLSTRNPCNFT-RVYGGKLOPTFNHNG 69
GmBRI1b     1 SGK-IAVNFLSGKTYVYIKNDG-SKECHGAGNLLEFAGISOOOLNRLSTRNPCNFT-RVYGGKLOPTFNHNG 69
GmBRL1a     1 AGL-VIPGRVSGKOFAFVRNEG-GTSCRGAGGLVEFEDIRTERLEGFPMVHSCPLT-RIYSGWTVYTFASNG 69
GmBRL1b     1 AGF-VIPGRVSGKOFAFVRNEG-GTSCRGAGGLVEFEDIRTERLEGFPMVHSCPLT-RIYSGRTVYTFASNG 69
GmBRL2a     1 LGAKSLFGILSGNTLVFVRNVG-N-SCKGVGGLLEFSGIRPERLLOVPTLRTCDFA-RLYSGPVLSQFTKYO 69
GmBRL2b     1 CGAKSLFGILSGNTLVFVRNVG-N-SCKGVGGLLEFSGIRPERLLOVPTLRTCDFT-RLYSGPVLS-FTKYO 68
Ps BRI1     1 SGK-VVVNFISGKTYVYIKNDG-SKECHGAGSLLEFAGINOEOLRRISTRNPCNFT-RVYGGKLOPTFTLNG 69
Os BRI1     1 SGK-MVVGLIVGRPYVYLRNDELSSECRGKGSLLEFTSIRPDDLSRMPSKKLCNFT-RMYVGSTEYTFNKNG 70
HvBRI1      1 SGK-MTVGLIIGRPYVYLRNDELSSOCRGKGSLLEFSSIRSEDLSRMPSKKLCNFT-RMYMGSTEYTFNKNG 70
Nt BRI1     1 SGN-IAVALLTGKRYVYIKNDG-SKECHGAGNLLEFGGIROEOLDRISTRHPCNFT-RVYRGITOPTFNHNG 69
Sl BRI1     1 SGN-IAVALLTGKRYVYIKNDG-SKECHGAGNLLEFGGIRQEQLDRISTRHPCNFT-RVYRGITQPTFNHNG 69

cons        1  *         : *.   .:::*      .*: * *.*:**  .*  :  *   ..   .  *  : *:* *      *     72
```

Figure S3. Alignment of the island domain sequences of the BR receptors from *Arabidopsis thaliana* (At), *Oryza sativa* (Os), *Glycine max* (Gm), *Solanum lycopersicum* (Sl), *Nicotiana tabacum* (Nt), *Pisum sativum* (Ps), and *Hordeum vulgare* (Hv). T-COFFEE V11.0 was used to construct the alignments [81]. The amino acid sequences that are identical in all aligned BR receptors are indicated with an asterisk (*), sequences that are conserved are indicated with a dot (. and :).

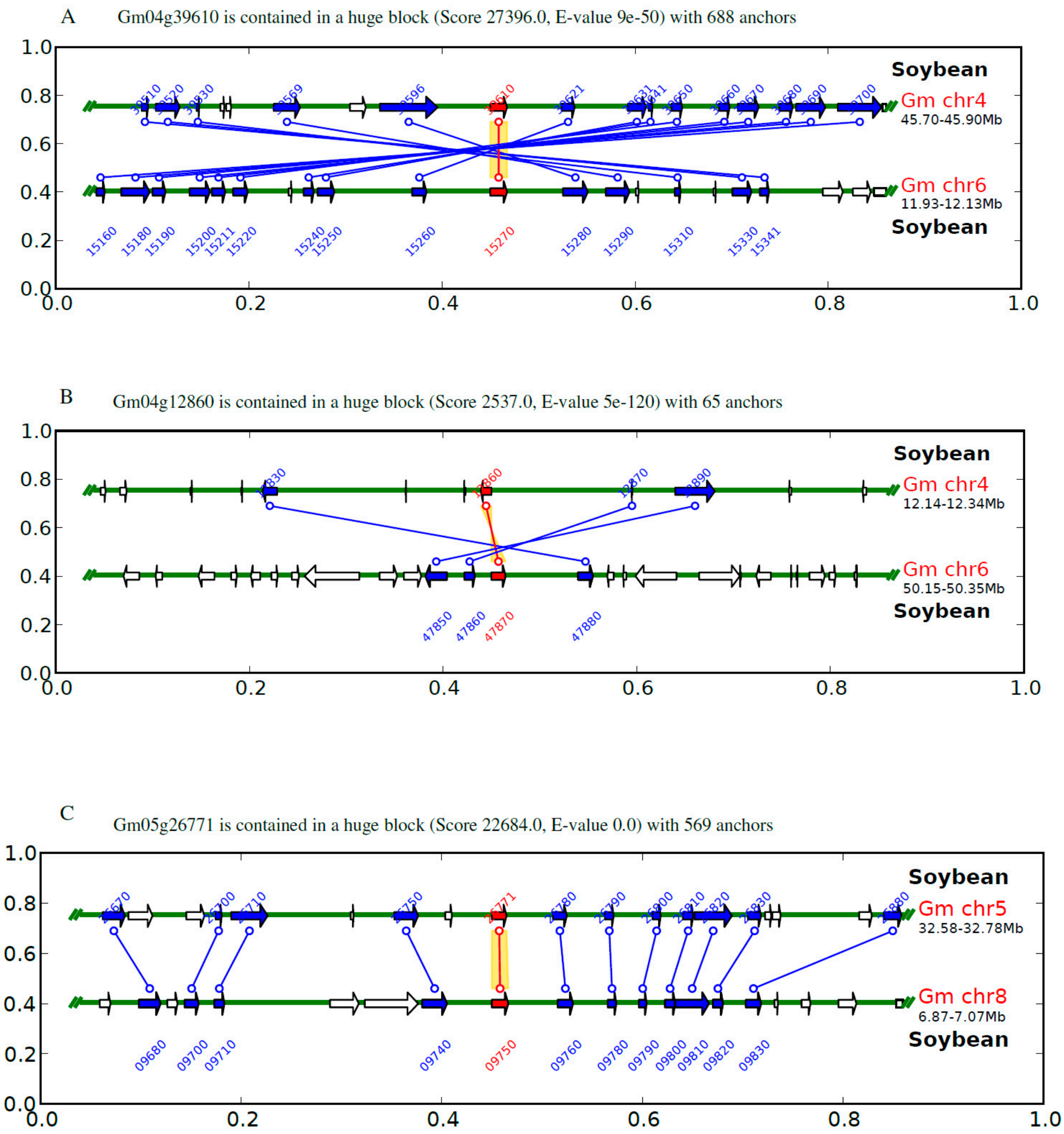

Figure S4. Duplication analysis of the BR receptor genes in soybean. Three gene duplication events can be found in soybean, *Glyma04g39610* (*GmBRI1b*) to *Glyma06g15270* (*GmBRI1a*) (**A**); *Glyma04g12860* (*GmBRL1a*) to *Glyma06g47870* (*GmBRL1b*) (**B**); and *Glyma05g26771* (*GmBRL2a*) to *Glyma08g09750* (*GmBRL2b*) (**C**). The intra-species blocks for *GmBRI1a*, *GmBRL1a*, and *GmBRL2a* are shown in A, B, and C, respectively. Graphs display 100-kb regions. Blue arrows indicate the other anchor genes in the region, red indicates the query locus.

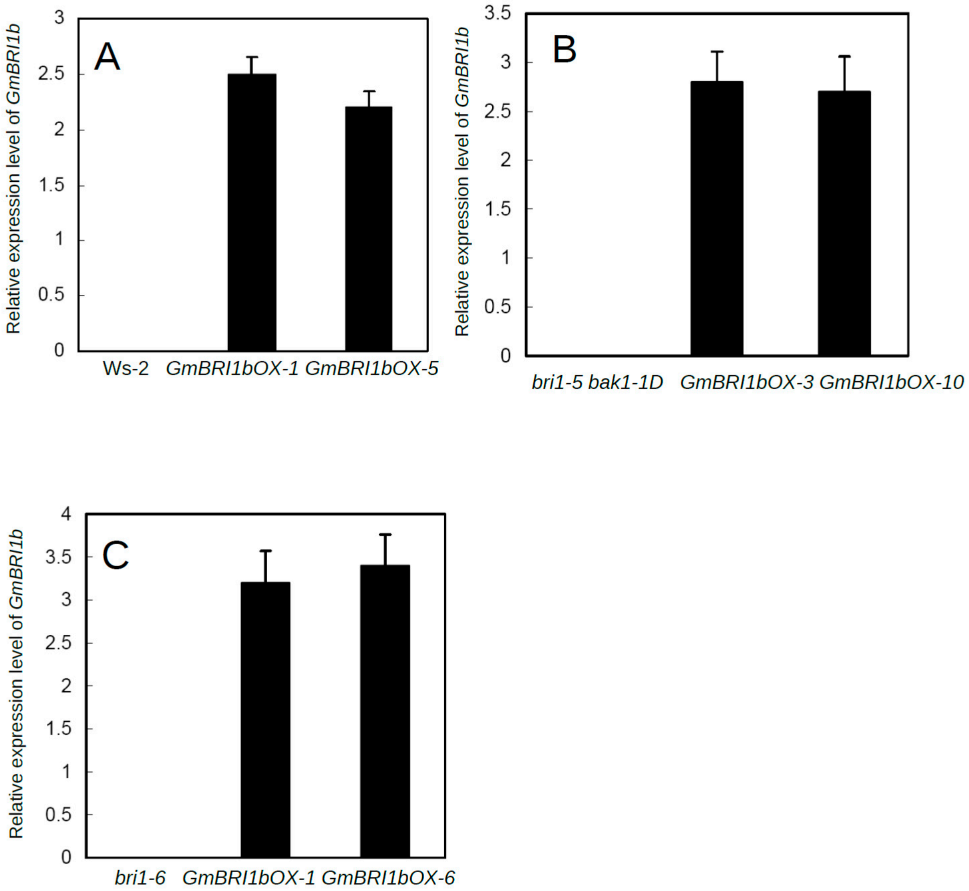

Figure S5. The relative expression levels of *GmBRI1b* in transgenic lines in Ws-2 (**A**), *bri1-5 bak1-1D* (**B**), and *bri1-6* (**C**) background, respectively. Quantitative real-time PCR was used to determine the relative transcription levels of *GmBRI1b* in transgenic lines. *AtEF-1a* was used to normalize the relative expression levels of *GmBRI1b*. Results are means ± SD from three independent experiments.
